# Spin in quantum chromodynamics 

**DOI:** 10.1126/sciadv.aeh1991

**Published:** 2026-09-16

**Authors:** Abhay Deshpande, Dmitri E. Kharzeev, Jinfeng Liao

**Affiliations:** ^1^ Center for Nuclear Frontiers in Nuclear Science, Department of Physics and Astronomy, Stony Brook University, New York 11794-3800, USA.; ^2^ Department of Physics, Brookhaven National Laboratory, Upton, NY 11973-5000, USA.; ^3^ Center for Nuclear Theory, Department of Physics and Astronomy, Stony Brook University, New York 11794-3800, USA.; ^4^ Energy and Photon Sciences Directorate, Condensed Matter and Materials Sciences Division, Brookhaven National Laboratory, Upton, NY 11973-5000, USA.; ^5^ Physics Department and Center for Exploration of Energy and Matter, Indiana University, 2401 N Milo B. Sampson Lane, Bloomington, IN 47408, USA.

## Abstract

This article surveys the role of spin in quantum chromodynamics (QCD), tracing its manifestations from the internal structure of the nucleon to the collective dynamics of strongly interacting matter. We review the decomposition of the proton spin into the spin and orbital angular momentum of quarks and gluons and discuss the theoretical and experimental progress that has shaped our modern understanding of polarized parton structure. We then examine spin transport phenomena in hot and dense QCD matter, highlighting theoretical developments of phase structures under rotation and spin hydrodynamics theory as well as the experimental discovery of global polarization and other related phenomena. Last, we present anomalous transport and the chiral magnetic effect as paradigmatic examples of how quantum anomalies convert chirality and topology into macroscopic, nondissipative currents whose signal can be measured in experiments. Together, these topics illustrate how spin provides a unifying framework linking hadronic structure, gauge field topology, quark field chirality, and relativistic many-body dynamics in QCD.

## INTRODUCTION

Over the past 100 years, spin has played a foundational role in the development of quantum theory, from the discovery of electron spin and the Pauli exclusion principle to the formulation of relativistic quantum mechanics and quantum field theory. In quantum chromodynamics (QCD), the non-Abelian gauge theory of quarks and gluons, spin is deeply intertwined with confinement, chiral symmetry, topology, and the collective behavior of strongly interacting matter. Over the past several decades, the study of spin in QCD has evolved from the nonrelativistic quark model to a broad and dynamic field encompassing polarized parton structure, spin transport in hot and dense matter, and anomaly-induced transport in relativistic plasmas.

This article surveys several interrelated aspects of spin in QCD. We begin with the spin structure of the nucleon, proceed to spin effects in QCD matter under extreme conditions, and culminate with the chiral magnetic effect (CME) and anomalous transport phenomena rooted in quantum anomalies. Together, these topics illustrate how spin serves as a unifying thread connecting hadronic structure, nonequilibrium quantum field theory, many-body transport phenomena, as well as macroscopic manifestations of topology, chirality, and anomaly.

### From the proton spin puzzle to QCD angular momentum decomposition

The modern era of QCD spin physics of the nucleon was catalyzed by the discovery, in polarized deep inelastic scattering (DIS) experiments by the European Muon Collaboration (EMC) (*1*), that quark helicities account for only a small fraction of the proton’s total spin $\frac{1}{2}$. This unexpected result, often referred to as the “proton spin crisis,” challenged the naive quark model picture in which the proton spin is simply the sum of the spins of its three valence quarks. Instead, it revealed the dynamical and many-body nature of angular momentum in QCD.

In field-theoretic terms, the proton spin arises from the expectation value of the QCD angular momentum operator **J**. Decomposing it into spin and orbital components of quark and gluon is subtle because of gauge invariance and the nonlocal structure of gauge fields. Various decompositions—notably those proposed by Ji (*2*) and by Jaffe and Manohar (*3*)—have clarified the interplay between gauge symmetry, partonic interpretation, and measurable observables such as generalized parton distributions (GPDs) (*4*–*6*) and transverse momentum–dependent distributions (TMDs) (*7*–*9*).

Experimentally, polarized lepton-nucleon scattering using fixed target experiments at CERN, SLAC, DESY, and JLab (*10*), polarized proton-proton collisions at the Relativistic Heavy Ion Collider (RHIC) at Brookhaven National Laboratory (BNL), and next-to-leading-order global QCD analyses have established that gluon helicity and orbital angular momentum (OAM) play essential roles in fulfilling the proton spin budget (*11*–*14*). The ongoing experimental program at facilities such as Jefferson Lab (*15*) and the future Electron-Ion Collider (EIC) (*16*) at BNL aims to map the multidimensional spin structure of the nucleon with unprecedented precision.

The spin of the nucleon thus emerges as a window into the internal dynamics of QCD: It probes the distribution of quark and gluon angular momentum, the role of confinement, and the structure of the QCD vacuum. It also provides the conceptual bridge to the broader theme of spin in QCD matter.

### Spin in hot and dense QCD matter

When nuclear matter is heated or compressed to extreme conditions, such as those achieved in relativistic heavy-ion collisions, QCD predicts a transition to a deconfined quark-gluon plasma (QGP). The experiments at RHIC and the Large Hadron Collider (LHC) have established that the QGP at experimentally accessible temperatures behaves as a strongly interacting fluid. In this fluid, spin becomes a dynamical variable coupled to the vorticity and magnetic fields in the QGP fluid.

One of the most notable recent discoveries in heavy-ion physics is the observation of global hyperon polarization, indicating that the QGP created in noncentral collisions has enormous vorticity. Measurements by the STAR Collaboration (*17*) have shown that Λ and $\bar{\Lambda }$ hyperons are polarized along the system’s angular momentum axis, providing the first direct evidence of a vortical relativistic fluid. This phenomenon links spin to relativistic hydrodynamics and local thermal equilibrium with finite angular momentum in a system governed by QCD.

Spin alignment of vector mesons, local spin polarization patterns, spin-dependent correlations, and possible local parity-odd domains further enrich the phenomenology. These observations demand a theoretical framework that incorporates spin degrees of freedom into relativistic kinetic theory and hydrodynamics. While the study of nucleon spin is a well-established field based on rigorous QCD theory, the connection from hot and dense QCD phenomena to QCD spin is relatively  recent and requires a deeper understanding of the underlying QCD dynamics. Recent developments in spin hydrodynamics and Wigner-function approaches have begun to establish such a framework, where spin transport arises naturally from gradients of temperature, chemical potential, and flow velocity. Thus, in QCD matter under extreme conditions, spin is not a passive quantum number but an active probe of the hot and dense medium’s structure and evolution.

### Anomalous transport and the CME

Perhaps the most profound connection between spin, topology, and macroscopic physics in QCD is embodied in anomalous transport phenomena. Quantum anomalies, such as the chiral anomaly, express the breakdown of classical symmetries at the quantum level due to ultraviolet quantum effects. In QCD, topologically nontrivial gluon configurations induce changes in chirality via the chiral anomaly, linking gauge-field topology to fermionic spin and helicity.

In the presence of a chiral imbalance and a magnetic field, this anomaly gives rise to the CME: the generation of an electric current along the direction of the magnetic field. This transport phenomenon is dissipationless and topological in origin, reflecting the spectral flow of chiral fermions in a magnetic field. It is a quintessential example of how microscopic quantum anomalies can manifest as macroscopic transport currents.

In heavy-ion collisions, intense magnetic fields are transiently generated, and topological fluctuations of the gluon fields can produce a local chirality imbalance. These conditions allow the CME to occur and induce a measurable signal. The search for the CME in heavy-ion experiments has motivated extensive theoretical and experimental efforts, including detailed measurements in the beam energy scan (BES) program at RHIC. While isolating the signal from backgrounds remains challenging, recent STAR results provide strong evidence for the CME (*18*). On the theory side, the broader framework of anomalous hydrodynamics and chiral kinetic theory has become a cornerstone of modern relativistic many-body physics.

Analogous phenomena have been observed in condensed matter systems such as Weyl and Dirac semimetals (*19*, *20*), where chiral quasiparticles and Berry curvature mimic the anomaly structure of high-energy QCD. This cross-disciplinary interplay underscores the universality of anomaly-induced transport.

### Spin as a unifying theme in QCD

Across these subfields—hadronic structure, strongly interacting matter, and anomalous transport—spin serves as a unifying concept. At the microscopic level, it encodes the helicity structure of quarks and gluons and the topology of gauge fields. At the macroscopic level, it governs polarization, vorticity, and nondissipative currents in relativistic fluids. The study of spin in QCD thus spans scales from the femtometer-scale structure of the proton to the collective dynamics of a quark-gluon plasma.

In this article, we aim to present a coherent narrative connecting these themes. We begin with the field-theoretic foundations of angular momentum in QCD and the phenomenology of the nucleon spin structure. We then explore spin transport effects in hot and dense QCD matter, emphasizing the interplay between polarization, vorticity, and hydrodynamics. Last, we discuss anomalous transport and the CME as paradigmatic examples of how quantum anomalies convert spin and chirality into observable macroscopic phenomena.

A century after the discovery of spin, QCD reveals that spin is not merely an intrinsic property of elementary particles but a dynamical, topological, and collective feature of strongly interacting matter. It may be noted that a complementary review of spin in fundamental interactions is provided by Budker *et al*. (*21*). 

## SPIN STRUCTURE OF THE NUCLEON IN QCD

### The proton spin problem

The question of how the spin of the nucleon emerges from the underlying dynamics of QCD has been central to strong-interaction physics for several decades. While the proton is a composite, relativistic, and strongly interacting bound state of quarks and gluons, it nevertheless carries a precisely defined spin quantum number, $J=\frac{1}{2}$. Understanding how this spin arises from quark and gluon degrees of freedom—through their intrinsic spins, orbital angular momenta, and correlations generated by confinement and chiral dynamics—has profoundly shaped modern hadron physics.

Early constituent quark models attributed the proton spin almost entirely to the alignment of quark spins. This simple picture was markedly challenged by polarized DIS experiments in the late 1980s (*1*), which revealed that quark helicities account for only a fraction of the nucleon spin. This so-called “spin crisis” triggered extensive experimental efforts around the world and intense associated theoretical activities aimed at uncovering the full spin decomposition of the nucleon within QCD.

In this section, we review the theoretical foundations, experimental discoveries, and contemporary understanding of nucleon spin structure in QCD. We emphasize the role of parton distributions, gauge invariance, OAM, as well as emerging insights from lattice QCD, GPDs, and quantum information–inspired perspectives.

### Spin decomposition in QCD

In QCD, the total angular momentum operator can be written schematically as

$$J^{i}=\frac{1}{2}\;\epsilon^{\mathrm{ijk}}\int d^{3}x\;\left[x^{j}\;T^{0k}\left(x\right)-x^{k}\;T^{0j}\left(x\right)\right]$$ (1)

where $T^{\mu \nu }$ is the energy-momentum tensor. Equivalently, this operator can be written in terms of the angular momentum tensor $M^{\mu \nu \rho }$

$$M^{\mu \nu \rho }\left(x\right)=x^{\nu }T^{\mu \rho }\left(x\right)-x^{\rho }T^{\mu \nu }\left(x\right),\;J^{\nu \rho }=\int d^{3}x\;M^{0\nu \rho }\left(x\right)$$ (2)

Here, $T^{\mu \nu }$ is understood to be the Belinfante-Rosenfeld improved, symmetric, and gauge-invariant energy-momentum tensor (*22*, *23*). With this choice, the total angular momentum tensor $M^{\mu \nu \rho }\left(x\right)$ takes a purely orbital form, with quark and gluon spin contributions implicitly absorbed into $T^{\mu \nu }$. Note that the Belinfante-Rosenfeld tensor is obtained from the canonical energy-momentum tensor by adding a total derivative of the spin current, $T_{\text{Bel}}^{\mu \nu }=T_{\text{can}}^{\mu \nu }+\frac{1}{2}\partial_{\lambda }\left(S^{\lambda \mu \nu }+S^{\mu \nu \lambda }+S^{\nu \mu \lambda }\right)$ which renders $T^{\mu \nu }$ symmetric while leaving all conserved charges unchanged.

A central challenge is to decompose **J** into physically meaningful quark and gluon contributions while maintaining gauge invariance and Lorentz covariance. We note that the separation of total angular momentum into spin and orbital components is, in general, sensitive to the choice of Lorentz frame. Two commonly discussed decompositions are as follows:

1) The canonical (Jaffe-Manohar) decomposition (*3*), which separates quark and gluon spin and OAM but is not manifestly gauge invariant.

2) The kinetic (Ji) decomposition (*2*), which is gauge invariant but combines gluon spin and orbital contributions.

In the Jaffe-Manohar decomposition, the nucleon spin is represented as

$$\frac{1}{2}=\frac{1}{2}\Delta \Sigma +\Delta G+L_{q}+L_{g}$$ (3)

where ΔΣ and ΔG are the quark and gluon helicity contributions, respectively, while $L_{q}$ and $L_{g}$ are the quark and gluon OAM, respectively. This form arises naturally in the parton model and is often used in the interpretation of high-energy scattering experiments.

In the Ji decomposition, the nucleon spin sum rule reads

$$\frac{1}{2}=\frac{1}{2}\Delta \Sigma +L_{q}^{\text{kin}}+J_{g}$$ (4)

where ΔΣ is the total quark helicity contribution, $L_{q}^{\text{kin}}$ the quark OAM, and $J_{g}$ the total gluon angular momentum. It may be noted that the $L_{q}^{\text{kin}}$ above is physically different from the $L_{q}$ term in Eq. 3.

### Quark helicity and the axial current

The helicity distribution of quarks inside the nucleon measures the difference between the probabilities to find quarks with spin aligned and antialigned with the longitudinal spin of the nucleon. In QCD, this quantity can be defined in a gauge-invariant way through the matrix element of the axial vector current; see (*11*, *24*) for reviews.

Let us consider a nucleon state ∣P,S〉 with four-momentum $P^{\mu }$ and covariant spin vector $S^{\mu }$, normalized as

$$S^{2}=-1,\;P\cdot S=0$$ (5)

The flavor–dependent axial current is defined as

$$J_{5\;q}^{\mu }\left(x\right)=\bar{q}\left(x\right)\gamma^{\mu }\gamma_{5}q\left(x\right)$$ (6)

where *q*(*x*) denotes the quark field of flavor *q*. The forward nucleon matrix element of this current defines the axial charge of the nucleon for that flavor

$$\langle P,S|\bar{q}\gamma^{\mu }\gamma_{5}q|P,S\rangle =2\;\Delta q\;S^{\mu }$$ (7)

The coefficient Δq is called the quark helicity contribution (or quark axial charge) for flavor *q*. Physically, it represents the net longitudinal polarization carried by quarks of that flavor in a longitudinally polarized nucleon.

This quantity has a direct partonic interpretation. In the infinite momentum frame, Δq can be written as the first moment of the polarized quark distribution function

$$\Delta q=\int_{0}^{1}\mathrm{dx}\left[\Delta q^{h}\left(x\right)+\Delta {\bar{q}}^{h}\left(x\right)\right]$$ (8)

where

$$\Delta q^{h}\left(x\right)=q^{↑}\left(x\right)-q^{↓}\left(x\right)$$ (9)

is the difference between the number densities of quarks with helicity aligned and antialigned with the helicity of the nucleon.

The connection between the operator definition and the partonic distribution can be made explicit through the light-cone correlation function

$$\Delta q^{h}\left(x\right)=\frac{1}{2}\int \frac{d\lambda }{2\pi }\;e^{i\lambda x}\langle P,S|\bar{q}\left(0\right)\gamma^{+}\gamma_{5}\;W\left(0,\lambda n\right)\;q\left(\lambda n\right)|P,S\rangle$$ (10)

where $n^{\mu }$ is a light-like vector satisfying $n^{2}=0$ and W(0,λn) is the Wilson line required for gauge invariance. Taking the first moment of this expression reproduces the axial current matrix element above. The $\Delta q^{h}\left(x\right)$ are the polarized parton distribution functions (PDFs).

Summing over quark flavors yields the total quark helicity contribution to the spin of the nucleon

$$\Delta \Sigma =\sum_{q}\Delta q=\sum_{q}\int_{0}^{1}\mathrm{dx}\;\left[\Delta q^{h}\left(x\right)+\Delta {\bar{q}}^{h}\left(x\right)\right]$$ (11)

Measurements from polarized DIS experiments have established that ΔΣ≈0.25 to −0.35 at typical DIS scales (see below for details), implying that most of the nucleon spin arises from gluon polarization and orbital motion. The scale dependence of ΔΣ is governed by the chiral anomaly and QCD evolution, reflecting the intrinsically relativistic and quantum nature of the problem.

A key theoretical ingredient in understanding the proton spin structure is the chiral anomaly (*25*, *26*). The divergence of the singlet axial current receives a gluonic contribution

$$\partial_{\mu }J_{5}^{\mu }=2{\mathrm{im}}_{q}\bar{q}\gamma_{5}q+\frac{\alpha_{s}}{4\pi }F_{\mu \nu }^{a}{\tilde{F}}^{\mu \nu a}$$ (12)

where $F_{\mu \nu }^{a}$ is the gluon field strength tensor and ${\tilde{F}}^{\mu \nu a}=\frac{1}{2}\epsilon^{\mu \nu \rho \sigma }F_{\rho \sigma }^{a}$ is its dual. Because of the anomaly, the first moment of the polarized structure function receives a contribution proportional to the gluon helicity (*27*–*30*). In certain factorization schemes, the measured quark spin fraction ${\Delta \Sigma }_{\text{meas}}$ can be written as

$${\Delta \Sigma }_{\text{meas}}=\Delta \Sigma -\frac{\alpha_{s}}{2\pi }\Delta G$$ (13)

This relation led to early speculation that a very large gluon polarization might explain the small value of the measured quark spin contribution. Subsequent experiments have shown that although ΔG is positive, it is not large enough to fully resolve the spin puzzle.

Polarized DIS has served as the primary probe of quark spin. In these experiments, a polarized lepton scatters from a polarized nucleon target. Both polarized muon and electron beams have been used to measure quark contributions to the nucleon spin.

The spin-dependent part of the cross section is described by the structure functions $g_{1}\left(x,Q^{2}\right)$ and $g_{2}\left(x,Q^{2}\right)$. At leading twist

$$g_{1}\left(x,Q^{2}\right)=\frac{1}{2}\sum_{q}e_{q}^{2}\left[\Delta q\left(x,Q^{2}\right)+\Delta \bar{q}(x,Q^{2})\right]+O\left(\alpha_{s}\right)$$ (14)

By extracting information on $\Delta q\left(x,Q^{2}\right)$ and $\Delta \bar{q}\left(x,Q^{2}\right)$ from experimental data, one can then use Eq. 11 to compute the total quark helicity contribution ΔΣ.

Major polarized DIS experiments include EMC (CERN), SMC (CERN), HERMES (DESY), COMPASS (CERN), SLAC experiments (E142, E143, E154, and E155), and a range of Jefferson Lab experiments; see (*11*) for review. CERN experiments used high-energy (190 to 240 GeV) polarized muon beams, while experiments at DESY, SLAC, and Jefferson Lab were performed with somewhat lower energy (8 to 27 GeV) electron/positron beams, resulting in measurements in complementary ($x-Q^{2}$) phase spaces. Next-to-leading-order global analysis of the data indicates

ΔΣ≈0.25−0.35 (15)

Thus, quark helicities account for roughly one-third of the proton spin. It should be emphasized that such contributions are dependent on the probing scale $Q^{2}$. All quoted results in the present subsection correspond to a typical scale $Q^{2}\approx 10\;\text{GeV}$.

Semi-inclusive DIS (SIDIS) allows the flavor decomposition of polarized PDFs by tagging hadrons in the final state. By combining SIDIS and inclusive DIS measurements, analyses indicate that Δu is positive and large, Δd is negative, and Δs is possibly negative (*11*).

### Gluon polarization

The gluon helicity contribution to the nucleon spin measures the net polarization of gluons inside a longitudinally polarized nucleon. Unlike the quark case, where the helicity is directly related to the matrix element of the local axial current, the definition of gluon helicity involves subtleties associated with gauge invariance.

In the parton model, the gluon helicity distribution is defined as

$$\Delta g\left(x\right)=g^{↑}\left(x\right)-g^{↓}\left(x\right)$$ (16)

where $g^{↑}\left(x\right)$ and $g^{↓}\left(x\right)$ denote the number densities of gluons with helicity aligned and antialigned with the helicity of the parent nucleon. The total gluon helicity contribution to the nucleon spin is given by the first moment of the polarized gluon distribution

$$\Delta G\left(Q^{2}\right)=\int_{0}^{1}\mathrm{dx}\;\Delta g\left(x,Q^{2}\right)$$ (17)

Here, $Q^{2}$ denotes the factorization scale, reflecting the scale dependence of parton distributions.

A gauge-invariant operator definition of the gluon helicity distribution can be formulated in terms of a light-cone correlation function of gluon field strength tensors

$$\Delta g\left(x\right)=\frac{i}{{\mathrm{xP}}^{+}}\int \frac{d\lambda }{2\pi }\;e^{i\lambda x}\langle P,S|F^{+\alpha }\left(0\right)\;W\left(0,\lambda n\right)\;{\tilde{F}}_{\alpha }^{+}\left(\lambda n\right)|P,S\rangle$$ (18)

Here, ${\tilde{F}}^{\mu \nu }$ is the dual field strength tensor, $n^{\mu }$ is a light-like vector ($n^{2}=0$), and W denotes the Wilson line ensuring gauge invariance. Unlike the quark axial charge, the gluon helicity does not correspond to the matrix element of a local gauge-invariant operator. However, in the light-cone gauge $A^{+}=0$, the first moment can be expressed as the nucleon matrix element of the operator

$$\Delta G=\frac{1}{2P^{+}}\langle P,S|\epsilon^{\mathrm{ij}}\;\text{Tr}\left[F^{+i}A^{j}\right]|P,S\rangle$$ (19)

which corresponds to the gluon spin operator in this gauge.

Experimentally, the gluon helicity distribution $\Delta g\left(x,Q^{2}\right)$ is constrained through several processes sensitive to polarized gluons, including high-$p_{T}$ hadron production and jet production in polarized proton-proton collisions, as well as scaling violations of the polarized structure function $g_{1}\left(x,Q^{2}\right)$ in DIS. Unlike quark helicities, ΔG enters polarized DIS only at higher order, making its experimental determination challenging.

Substantial progress has been achieved through polarized proton-proton collisions, notably via measurements of double-spin asymmetries in jet, hadron, and, most recently, direct photon production. Data indicate a positive gluon polarization in the measured mid-*x* range, as discussed below. Because of a lack of data, the contributions from small *x* remain largely unknown yet could potentially be substantial.

At small Bjorken *x*, gluon densities grow rapidly, and nonlinear QCD dynamics may become important. Understanding the spin structure in this regime requires extending helicity evolution equations (*31*–*33*) and may involve nontrivial phenomena related to gluon saturation (*34*, *35*) and the Color Glass Condensate (*36*, *37*). The future EIC is expected to play a decisive role in resolving the small-*x* contribution to gluon spin and consequently to OAM (*16*) as well, which we will discuss below.

### Orbital angular momentum

#### Generalized parton distributions

GPDs provide a unified framework that interpolates between ordinary PDFs and the elastic form factors of the nucleon. See (*4*, *5*) for reviews. They encode correlated information about the longitudinal momentum and the transverse spatial distribution of quarks and gluons inside the nucleon and therefore allow one to access a three-dimensional picture of nucleon structure.

GPDs arise in the description of hard exclusive processes such as deeply virtual Compton scattering (DVCS) (*38*)

$$\gamma^{*}p\to \gamma p$$

and deeply virtual meson production. These reactions are characterized by a large photon virtuality $Q^{2}$ and a small momentum transfer *t* to the nucleon. In the Bjorken regime

$$Q^{2}\gg \Lambda_{\text{QCD}}^{2},\;x_{B}\;\text{fixed}$$

QCD factorization allows the amplitude to be written as a convolution of a perturbatively calculable coefficient function with nonperturbative GPDs. The relevant kinematic variables include the following: *x*, the average longitudinal momentum fraction carried by the struck parton; ξ, the longitudinal momentum transfer (“skewness”), related to the Bjorken variable as $\xi ≃\frac{x_{B}}{2-x_{B}}$; and $t={\left(p^{\prime }-p\right)}^{2}$, the invariant momentum transfer to the nucleon.

GPDs are defined through off-forward matrix elements of light-cone bilocal operators. For quarks, one considers

$${\int \frac{{\mathrm{dz}}^{-}}{4\pi }\;e^{{\mathrm{ixP}}^{+}z^{-}}\langle p^{\prime },s^{\prime }|\bar{\psi }\left(-\frac{z}{2}\right)\gamma^{+}\psi \left(\frac{z}{2}\right)|p,s\rangle |}_{z^{+}=0,\;z_{⊥}=0}$$

where $P=\left(p+p^{\prime }\right)/2$. The most general parametrization consistent with Lorentz invariance yields

$$\begin{array}{c} \int \frac{{\mathrm{dz}}^{-}}{4\pi }\;e^{{\mathrm{ixP}}^{+}z^{-}}\langle p^{\prime },s^{\prime }|\bar{\psi }\left(-\frac{z}{2}\right)\gamma^{+}\psi \left(\frac{z}{2}\right)|p,s\rangle \\ =\frac{1}{2P^{+}}\bar{u}\left(p^{\prime },s^{\prime }\right)\left[\gamma^{+}H^{q}\left(x,\xi ,t\right)+\frac{i\sigma^{+\mu }\Delta_{\mu }}{2M}E^{q}(x,\xi ,t)\right]u\left(p,s\right) \end{array}$$ (20)

where $\Delta =p^{\prime }-p$. The functions $H^{q}$ and $E^{q}$ are the quark GPDs. Analogous distributions exist for gluons.

In addition to *H* and *E*, polarized quark operators define two further GPDs

$${\tilde{H}}^{q}\left(x,\xi ,t\right),\;{\tilde{E}}^{q}\left(x,\xi ,t\right)$$

GPDs interpolate between well-known quantities. In the forward limit

$$p^{\prime }\to p\;\left(\xi \to 0,t\to 0\right)$$

the distributions reduce to ordinary parton densities

$$H^{q}\left(x,0,0\right)=q\left(x\right),\;{\tilde{H}}^{q}\left(x,0,0\right)=\Delta q\left(x\right)$$

The first moments of GPDs reproduce nucleon form factors

$$\int_{-1}^{1}\mathrm{dx}\;H^{q}\left(x,\xi ,t\right)=F_{1}^{q}\left(t\right)$$ (21)

$$\int_{-1}^{1}\mathrm{dx}\;E^{q}\left(x,\xi ,t\right)=F_{2}^{q}\left(t\right)$$ (22)

Thus, GPDs connect inclusive measurements, which probe parton distributions, with elastic scattering that measures nucleon form factors.

One of the most important results involving GPDs is the Ji sum rule, which relates the total angular momentum carried by quarks to the second moment of the GPDs *H* and *E*

$$J_{q}=\frac{1}{2}\int_{-1}^{1}\mathrm{dx}\;x\left[H^{q}\left(x,\xi ,0\right)+E^{q}(x,\xi ,0)\right]$$ (23)

The right-hand side is independent of ξ. This relation could potentially allow the extraction of the total quark angular momentum $J_{q}$ from measurements of GPDs, subject to the experimental constraints on the accessible range of *x* at fixed ξ. Combining it with the quark spin contribution ΔΣ obtained from polarized DIS would then give access to the quark OAM

$$L_{q}=J_{q}-\frac{1}{2}\Delta \Sigma$$

An analogous relation exists for gluons

$$J_{g}=\int_{0}^{1}\mathrm{dx}\;x\left[H^{g}\left(x,\xi ,0\right)+E^{g}(x,\xi ,0)\right]$$ (24)

GPDs are primarily accessed through hard exclusive processes. The most important of these is DVCS, where the interference between the DVCS amplitude and the Bethe-Heitler process allows one to extract Compton form factors that are related to GPDs.

Measurements of DVCS have been carried out at HERA, Jefferson Lab, and COMPASS, providing increasingly precise constraints on GPDs. The upgraded 12 GeV program at Jefferson Lab and the future EIC will greatly extend the kinematic reach and precision of these measurements, enabling a quantitative determination of the partonic contributions to the nucleon spin through Eq. 23. Beyond their role in spin decomposition, GPDs allow one to construct transverse spatial distributions of partons through Fourier transformations with respect to the momentum transfer *t*, leading to a tomographic picture of the nucleon.

#### Wigner distributions and OAM

A particularly illuminating framework for understanding the orbital motion of partons inside the nucleon is provided by phase-space distributions; see (*39*) for a review. In quantum mechanics, the closest analog to a classical phase-space distribution is the Wigner distribution. In the context of QCD, partonic Wigner distributions encode the joint dependence of partons on their transverse position and transverse momentum, providing a five-dimensional description of the nucleon structure (*40*).

For quarks, the gauge-invariant Wigner distribution can be defined through the Fourier transform of a nonlocal quark correlator

$$\begin{array}{c} W^{\left[\Gamma \right]}\left(x,k_{T},b_{T}\right)=\int \frac{d^{2}\Delta_{T}}{{\left(2\pi \right)}^{2}}\;e^{-i\Delta_{T}\cdot b_{T}}\;\int \frac{{\mathrm{dz}}^{-}d^{2}z_{T}}{{\left(2\pi \right)}^{3}}\;e^{{\mathrm{ixP}}^{+}z^{-}-ik_{T}\cdot z_{T}}\\ \langle P+\frac{\Delta }{2},S|\bar{q}\left(-\frac{z}{2}\right)\Gamma \;W\;q\left(\frac{z}{2}\right)|P-\frac{\Delta }{2},S\rangle \end{array}$$ (25)

Here, *x* is the longitudinal momentum fraction carried by the quark, $k_{T}$ its transverse momentum, and $b_{T}$ the transverse position (impact parameter) relative to the nucleon center. The matrix Γ selects the quark polarization structure, and W denotes the Wilson line required for gauge invariance.

The Wigner distribution therefore depends simultaneously on $\left(x,k_{T},b_{T}\right)$ and provides a phase-space picture of the nucleon in terms of quark degrees of freedom. However, because of the uncertainty principle, Wigner distributions cannot be interpreted as classical probability densities and may take negative values.

These distributions are closely related to generalized TMDs (GTMDs). GTMDs arise as the momentum-space representation of the same quark correlator before the Fourier transform with respect to the transverse momentum transfer $\Delta_{T}$. They therefore contain the most complete information about the partonic structure of the nucleon.

A key application of Wigner distributions is the description of OAM carried by quarks. In a semiclassical picture, the OAM corresponds to the expectation value of the phase-space operator $b_{T}\times k_{T}$. The quark OAM can therefore be expressed as the following phase-space integral

$$L_{q}=\int \mathrm{dx}\;d^{2}k_{T}\;d^{2}b_{T}\;{\left(b_{T}\times k_{T}\right)}_{z}\;W^{\left[\gamma^{+}\right]}\left(x,k_{T},b_{T}\right)$$ (26)

This relation provides an intuitive interpretation of partonic orbital motion: The angular momentum arises from the correlation between the transverse position and transverse momentum of partons inside the nucleon. Depending on the choice of gauge link in the correlator, the resulting OAM corresponds either to the canonical OAM [Jaffe-Manohar decomposition (*3*)] or to the kinetic OAM appearing in the Ji decomposition (*2*).

The phase-space formulation in terms of Wigner distributions therefore provides a unified and intuitive picture of OAM in QCD. It connects the geometric interpretation of parton motion inside the nucleon with measurable quantities such as GPDs and TMDs and plays an important role in modern theoretical and phenomenological studies of the nucleon spin structure.

### Lattice QCD results on the nucleon’s spin

Lattice QCD provides a first-principles framework for determining the spin decomposition of the nucleon directly from the QCD path integral; see (*41*) for an overview. In practice, lattice calculations access these quantities through matrix elements of local operators, which makes the Ji decomposition suitable for Euclidean simulations (*2*).

The quark spin contribution is obtained from the forward nucleon matrix element of the axial current (Eq. 7), and hence $\Delta \Sigma =\sum_{q}\Delta q$. Modern lattice calculations include not only the connected quark-line contributions but also the disconnected diagrams, which are numerically demanding but essential for the flavor-singlet combination. A notable development in this direction has been the use of the anomalous Ward identity, which provides an important cross-check on the flavor-singlet axial channel (*42*). Calculations with controlled continuum, chiral, and finite-volume systematics now consistently find that quark spin accounts for only a fraction of the proton spin, in qualitative agreement with polarized DIS and global analyses (*43*, *44*).

The total angular momentum carried by quarks and gluons is extracted from the nucleon matrix elements of the QCD energy-momentum tensor. For quarks

$$J_{q}=\frac{1}{2}\left[A_{q}\left(0\right)+B_{q}\left(0\right)\right]$$ (27)

where $A_{q}\left(t\right)$ and $B_{q}\left(t\right)$ are the generalized form factors of the quark energy-momentum tensor, evaluated at zero momentum transfer. An analogous relation holds for the gluon contribution

$$J_{g}=\frac{1}{2}\left[A_{g}\left(0\right)+B_{g}\left(0\right)\right]$$ (28)

Once $J_{q}$ and ΔΣ are known, the kinetic quark OAM follows from

$$L_{q}=J_{q}-\frac{1}{2}\Delta \Sigma$$ (29)

This program has been realized in several state-of-the-art simulations, including calculations at or near the physical pion mass and with nonperturbative renormalization of both quark and gluon operators. These studies indicate that a substantial part of the proton spin resides in quark orbital motion and in gluonic degrees of freedom (*44*, *45*).

The gluon sector is more challenging because of operator mixing and the noisy nature of gluonic observables. Nevertheless, lattice QCD has made important progress. Early work determined the glue spin in a finite-momentum frame and connected it to the gluon helicity through large-momentum effective theory ideas (*46*). More recent calculations of the gluon energy-momentum tensor and of proton gravitational form factors have provided increasingly robust information on the gluon contribution to the proton momentum and total angular momentum (*44*, *45*).

Recent developments also extend lattice access beyond integrated quantities. In particular, calculations of moments of axial-vector GPDs provide a more differential picture of how quark helicity and OAM are distributed and offer a direct bridge between lattice QCD and the GPD-based description of nucleon spin structure (*47*). This is an important step toward a fully multidimensional, tomographic understanding of the nucleon spin.

Overall, the current lattice-QCD picture is that the quark helicity contribution is considerably smaller than the total proton spin, while the remaining spin is shared between quark OAM and gluonic contributions. Although systematic uncertainties in the gluon sector and in the separation of individual components remain under active study, lattice QCD has by now established itself as an essential nonperturbative tool for the quantitative decomposition of the nucleon spin (*48*).

### Experimental status and global analyses

This section briefly reviews the current experimental status of nucleon spin studies, emphasizing the contributions of quark spin, gluon spin, and OAM, as well as the emerging picture of three-dimensional nucleon structure; see (*11*) for a detailed overview.

#### Quark spin contribution

Polarized DIS has served as the primary probe of quark spin in which a polarized lepton scatters from a polarized nucleon target. Both polarized muon and electron beams have been used to measure quark contributions to the nucleon spin. SIDIS allows the flavor decomposition of polarized PDFs by tagging hadrons in the final state; see, e.g., (*49*, *50*).

#### Gluon spin contribution

The gluon helicity distribution Δg(x) contributes to the evolution of polarized structure functions and can be measured through processes sensitive to gluons. The integrated gluon contribution is

$$\Delta G\left(Q^{2}\right)=\int_{0}^{1}\mathrm{dx}\;\Delta g\left(x,Q^{2}\right)$$ (30)

Experimental probes include double longitudinal lepton-nucleon and proton-proton spin asymmetry measurements. The following measurements have resulted in our current knowledge of the polarized gluon distribution: scaling violation of $g_{1}\left(x,Q^{2}\right)$ in polarized DIS; photon-gluon fusion in DIS; high-$p_{T}$ hadron production; and heavy flavor, jet, and pion production in polarized proton-proton collisions.

Polarized proton collisions at the RHIC have provided the most precise constraints on gluon spin yet. Measurements were performed at three different center-of-mass energies: 62.4, 200, and 510 GeV (*51*–*57*). Experiments measure the longitudinal double-spin asymmetry

$$A_{\text{LL}}=\frac{\sigma^{++}-\sigma^{+-}}{\sigma^{++}+\sigma^{+-}}$$ (31)

#### Orbital angular momentum

Since quark and gluon spins do not fully account for the nucleon spin, OAM must play a major role. Direct measurement of OAM is challenging, but information can be extracted through GPDs.

GPDs are accessed experimentally through exclusive reactions such as DVCS

ep→epγ (32)

Major experimental programs include HERMES, COMPASS, and Jefferson Lab. Jefferson Lab’s 12 GeV upgrade has substantially improved the precision of DVCS measurements.

#### Transverse spin and TMDs

A complementary description of nucleon spin structure is provided by TMDs; see (*8*) for a review. Important examples include the Sivers function (*58*), the Boer-Mulders function (*59*, *60*), and the transversity distribution (*61*, *62*). These quantities encode correlations between parton transverse momentum and spin. Measurements of TMDs have been performed in SIDIS experiments at HERMES, COMPASS, and Jefferson Lab (*63*–*66*).

#### Spin structure at small x

At very small Bjorken *x*, gluons dominate the nucleon wave function, and nonlinear QCD dynamics become important. The helicity distributions evolve according to modified small-*x* evolution equations that resum double logarithms of energy.

Recent theoretical developments predict that polarized parton distributions grow rapidly at small *x*, implying that a considerable fraction of the nucleon spin may reside in the currently unexplored region $x<10^{-3}$ (*31*–*33*). Accessing this region is one of the primary goals of the EIC.

#### Global analyses of polarized parton distributions

The most precise information about the spin structure of the nucleon comes from global QCD analyses that extract polarized PDFs by combining data from a wide range of experiments. Modern global fits incorporate next-to-leading-order QCD evolution and treat experimental uncertainties using sophisticated statistical methods. The most widely used analyses are performed by the DSSV collaboration (*67*–*69*), the JAM collaboration (*70*–*72*), and the NNPDFpol collaboration (*73*). Although these analyses use somewhat different methodologies, their results are broadly consistent and provide a coherent picture of the nucleon spin decomposition. We summarize their findings in Table 1.

**Table 1.** Summary of results from major global analyses of polarized PDFs at a typical scale *Q*^2^ ≈ 10 GeV^2^. Here, Δ*u*, Δ*d*, and Δ*s* denote the flavor-specific contributions as defined in Eq. 8. The gluon moment is quoted only for the region *x* > 0.01 where experimental constraints from RHIC are available.

Global fit	ΔΣ	Δ*G*(*x* > 0.01)	Δ*u*	Δ*d*	Δ*s*
DSSV14	0.27 ± 0.04	$0.20_{-0.07}^{+0.06}$	0.83 ± 0.03	−0.44 ± 0.03	−0.03 ± 0.01
JAM20	0.26 ± 0.03	0.21 ± 0.10	0.84 ± 0.02	−0.43 ± 0.02	−0.05 ± 0.02
NNPDFpol1.1	0.28 ± 0.05	0.23 ± 0.09	0.82 ± 0.05	−0.45 ± 0.05	−0.04 ± 0.03

Combining the results of global analyses, the present experimental picture of the proton spin decomposition can be summarized as

$$\frac{1}{2}\Delta \Sigma \approx 0.13$$ (33)

ΔG(x>0.01)≈0.20 (34)

$$L_{q}+L_{g}+\Delta G\left(x\leq 0.01\right)\approx 0.17$$ (35)

Thus, quark helicities account for roughly one-quarter of the proton spin, gluon helicities contribute a comparable fraction in the currently measured region, and the remainder must arise from OAM of quarks and gluons, as well as from gluon helicity at small-*x*. See Table 1 that summarizes the results of the global fits. Note that all these estimates are dependent on probe scale, and the quoted ranges are for a typical scale $Q^{2}\approx 10\;{\text{GeV}}^{2}$. A complete understanding of this decomposition will require measurements extending to smaller Bjorken *x*, as well as improved constraints on GPDs and TMDs.

### Future prospects: The EIC

In the near future, ongoing efforts such as the JLab 12 GeV program and the CERN COMPASS/AMBER program will continue advancing this field. In the longer term, the EIC will revolutionize the study of nucleon spin structure. The EIC will provide polarized electron and proton beams, high luminosity, and access to small Bjorken *x*.

Key goals of the EIC include:

1) determining the small-*x* gluon helicity distribution, 

2) mapping three-dimensional nucleon structure, 

3) precision measurements of TMDs, 

4) improved constraints on OAM, and 

5) understanding the role of gluon saturation. 

**Fig. 1.**
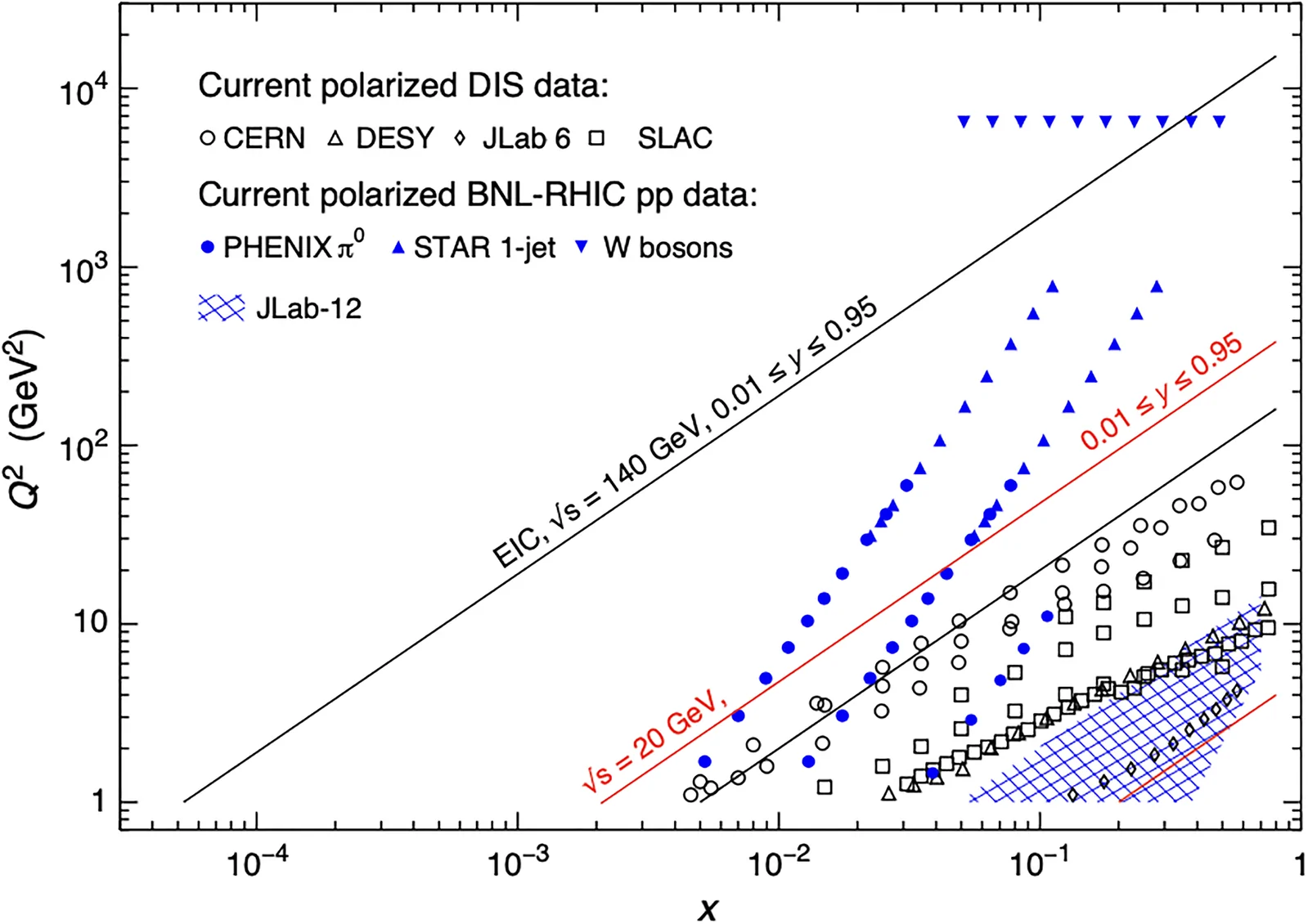
The $x-Q^{2}$ coverage of the EIC for two different center-of-mass energies. This is compared with polarized ep fixed target experimental measurements at CERN, DESY, Jefferson Lab, and SLAC, as well as pp experiments at RHIC.

**Fig. 2.**
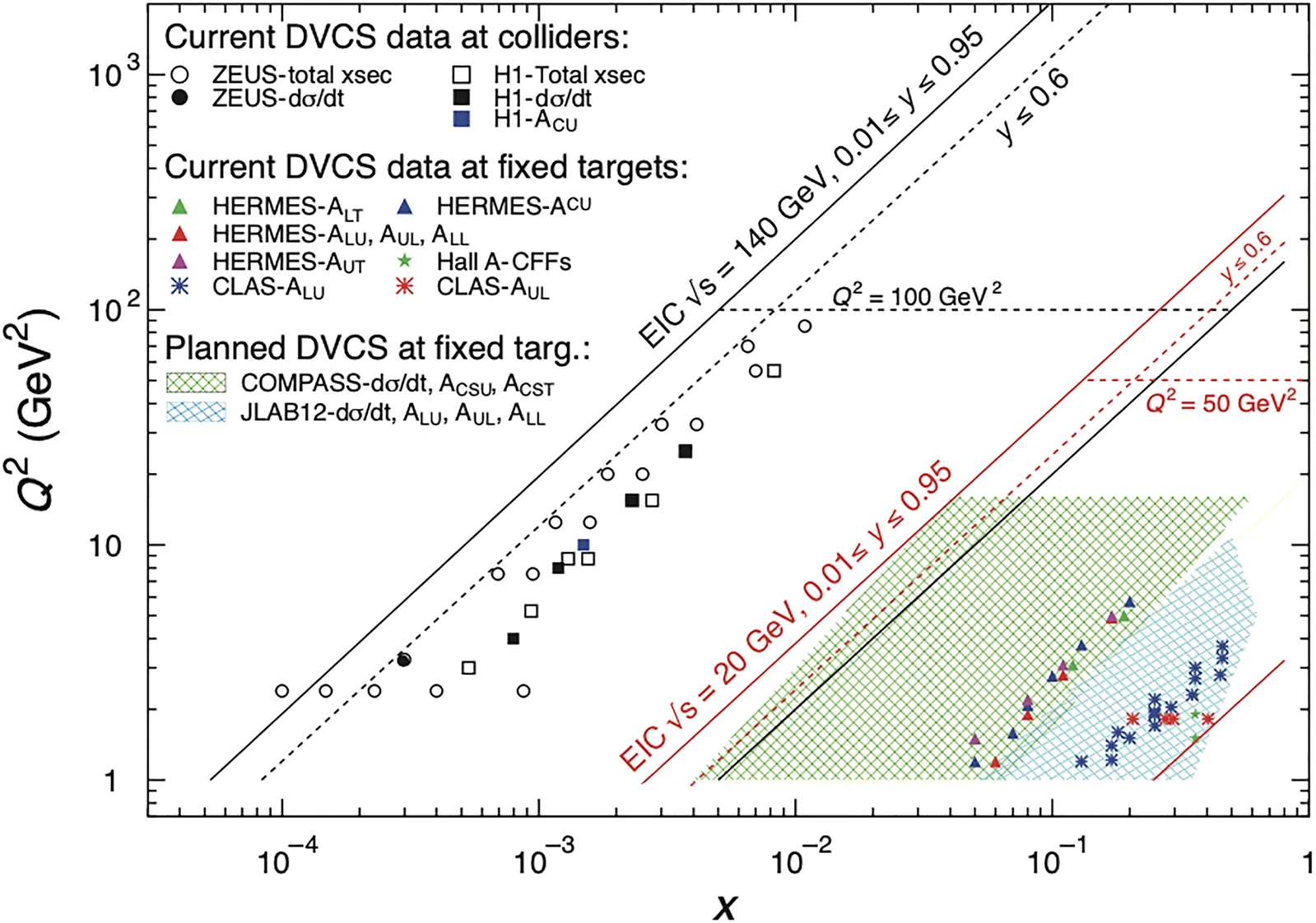
The kinematic coverage of the EIC for the DVCS process. This is compared with other fixed-target and collider-based DVCS experiments at CERN, JLab, and HERA.

**Fig. 3.**
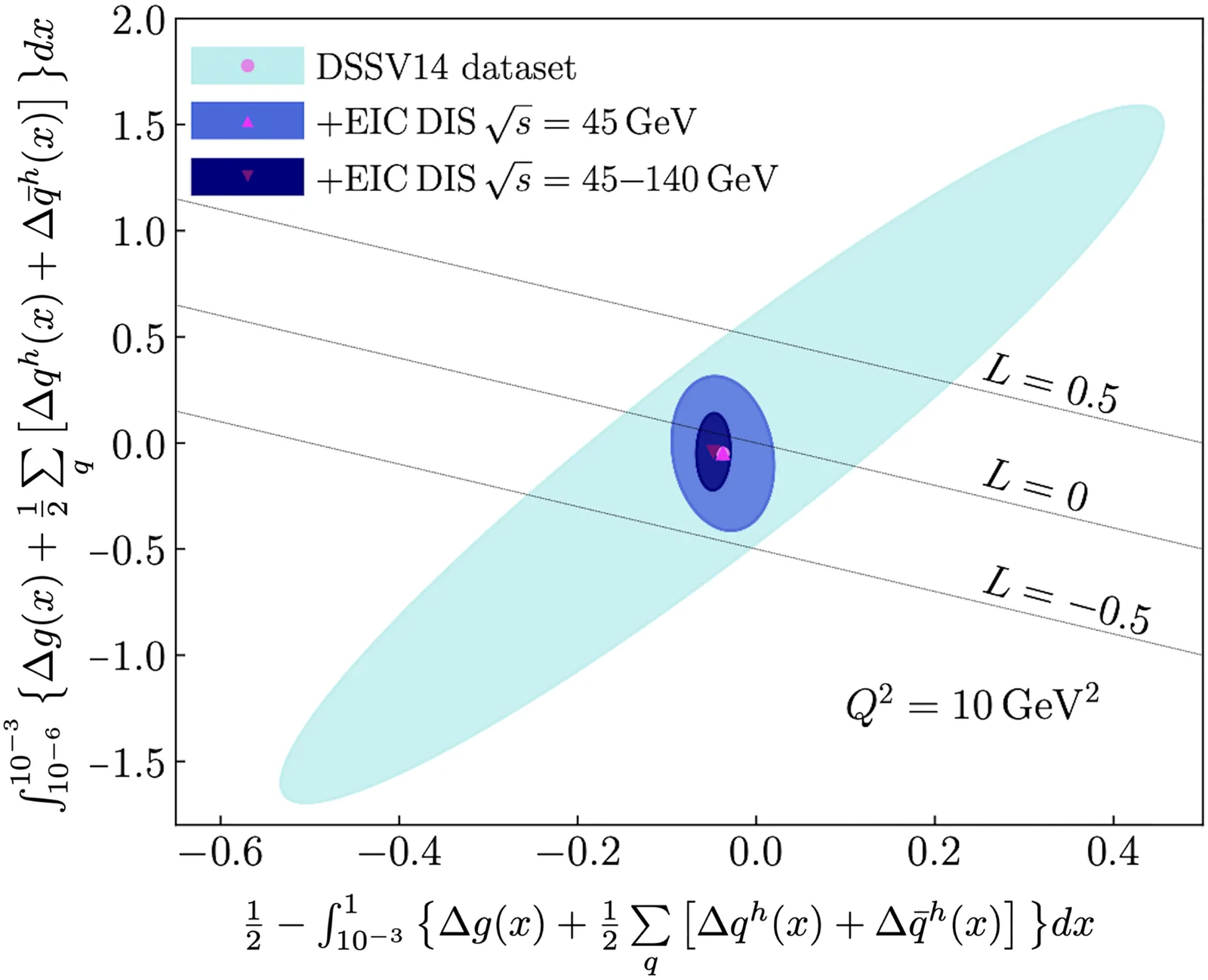
Estimated OAM contributions to the proton spin at *Q*^2^ = 10 GeV^2^, using the existing data and future EIC measurements. The horizontal axis shows the difference between proton spin (1/2) and the contribution from the spin of quarks and gluons for a momentum fraction down to *x* = 0.001. The vertical axis presents the spin contribution from partons with momentum fractions between 10^−6^ and 10^−3^. The ellipses correspond to the 1σ correlated uncertainty for the DSSV14 dataset (light blue), the fit including EIC $\sqrt{s_{\text{NN}}}$ = 45-GeV pseudodata (blue), and the reweighing with $\sqrt{s_{\text{NN}}}$ = 140-GeV pseudodata.

In Fig. 1, we illustrate the planned kinematical coverage in Bjorken *x* and $Q^{2}$ of the EIC in comparison to the current and previous facilities. It can be seen that the EIC will enable a marked extension of the previously accessible kinematic domain, which should allow a much more complete understanding of the proton’s spin decomposition. In particular, a marked improvement will be made in the study of the DVCS, as illustrated in Fig. 2. The EIC measurements, combined with currently available experimental data, will allow precise determination of the OAM contributions to the proton’s spin, as illustrated in Fig. 3.

### Summary

Decades of experimental and theoretical work have greatly advanced our understanding of nucleon spin. Current evidence indicates that:

1) quark helicities contribute roughly one-third of the nucleon spin,

2) gluon helicities (in the currently accessible *x* > 0.01 region) provide a moderate positive contribution, and

3) OAM plays an essential role.

Key open questions include the precise role of gluons and OAM at small *x*, the interplay between spin and confinement, and the unification of partonic and quantum information–based descriptions (*74*–*76*). Beyond the partonic picture, the nucleon spin problem highlights the deeply quantum nature of hadron structure. Spin correlations reflect entanglement among quarks and gluons confined within the proton.

Upcoming experiments at the EIC, continued advances in analytical methods and lattice QCD, and cross-fertilization with quantum information theory promise to deepen our understanding of how a simple quantum number—spin—emerges from the complex, many-body dynamics of QCD.

## SPIN DYNAMICS IN STRONGLY INTERACTING MATTER

In relativistic systems, the spin and orbital components are intrinsically entangled within the total angular momentum which, like energy and momentum, is an exactly conserved quantity that arises from fundamental spacetime symmetries. Hence, it is an important degree of freedom, akin to energy and momentum, for determining and characterizing the structures and properties of matter. The interplay between nonzero angular momentum and microscopic QCD dynamics can be highly nontrivial—the nucleon with $\frac{\hbar }{2}$ angular momentum is a perfect example, as discussed in the previous section. However, the role of angular momentum in strongly interacting matter (i.e., QCD many-body systems) has received relatively little attention until recently. Thanks to the latest discoveries from heavy-ion collisions, this new degree of freedom and the induced spin dynamics have been met with broad enthusiasm by the community. There are now diverse lines of inquiry into aspects of strongly interacting matter with angular momentum, which we aim to convey in the rest of this section. The first three subsections will focus on the conceptual understanding of how angular momentum (or rotation) influences the properties of matter both in and out of thermal equilibrium. The last subsection will then discuss how a key theoretical prediction was experimentally found in heavy-ion collisions as well as how it led to many subsequent developments.

### Rotational polarization of spin

The quantum nature of spin is best revealed in its polarizability: When “measured” (in a broad sense) by an external probe (like a magnetic field **B**) along a certain direction, it becomes aligned with the probe either in a parallel or antiparallel way. For a spin-$\frac{1}{2}$ particle (such as an electron), Pauli famously characterized this property as a “classically indescribable two-valuedness” around 1924. Physicists first came to appreciate this salient feature of spin in atomic spectrum lines via the Zeeman effect and Paschen-Back effect. The magnetic polarization of spin is also behind the Pauli paramagnetism in many-body systems.

However, there was a nice prelude long before the formal discovery of spin, namely, the Einstein–de Haas effect found in 1915 (*77*), where a solid sample was shown to start rotation after a change in its magnetization. This clearly demonstrated the link between the sample’s magnetic moment and its angular momentum, i.e., Δ**M** → Δ**J**, though the role of spin was not explicitly understood. Immediately afterward, a physicist named Barnett found the “mirror” effect (*78*, *79*) also in 1915: Rotating a solid sample results in a change of its magnetic moment, or Δ**J** → Δ**M**. What is more, Barnett managed to measure correctly the so-called gyromagnetic ratio *g* ≈ 2, the first confirmation of spin as a distinct and dominant source of magnetism.

The underlying mechanism of the Barnett effect can be understood as the rotational polarization of spin. When one increases the global rotation of the sample, the spin-orbital coupling ultimately transfers a certain fraction of the added angular momentum into the spin component, causing the particles’ spins to align preferably along the direction of the angular momentum. This is essentially an “equipartition” of angular momentum, distributing the total across all degrees of freedom. Such a rotational polarization can be described, in close analogy to magnetic polarization, as an effective coupling between a microscopic particle and macroscopic rotation in a matter system

$$H_{R}=-\omega \cdot j=-\omega \cdot \left(l+s\right)\to \rho \left(l,s\right)\propto e^{\omega \cdot \left(l+s\right)/T}$$ (36)

where **l** and **s** are the OAM and spin of the microscopic particle, and ω is the rotational angular velocity associated with the system’s total angular momentum (in exactly the same way as temperature or chemical potential being associated with the total energy or net charge). ρ(**l**, **s**) denotes the probability density of states with given OAM **l** and spin **s**. For a many-body system in thermal equilibrium at temperature *T*, one anticipates a Boltzmann factor for spin degrees of freedom $∼e^{\omega \cdot s/T}$, resulting in a macroscopic polarization with more spins pointing along the rotation axis than against it.

For a rotating rigid body such as the solid samples used by Einstein–de Haas and by Barnett, the connection between its angular momentum and rotational angular velocity is relatively straightforward. This issue becomes tricky for compressible fluids, as most relativistic fluids are. In such systems, rotation manifests itself locally as a fluid vorticity field ω(x) which can exhibit complex patterns but is nevertheless amenable to polarize spins as in Eq. 36. There are active investigations in condensed matter physics that use fluid vorticity to control electron spin transport, aka “fluid spintronics” (*80*, *81*). When applied to systems with chiral fermions, such rotational polarization leads to nontrivial phenomena like the chiral vortical effect (*82*, *83*) and could play interesting roles in astrophysical processes (*84*).

### Phases of QCD matter under rotation

**Fig. 4.**
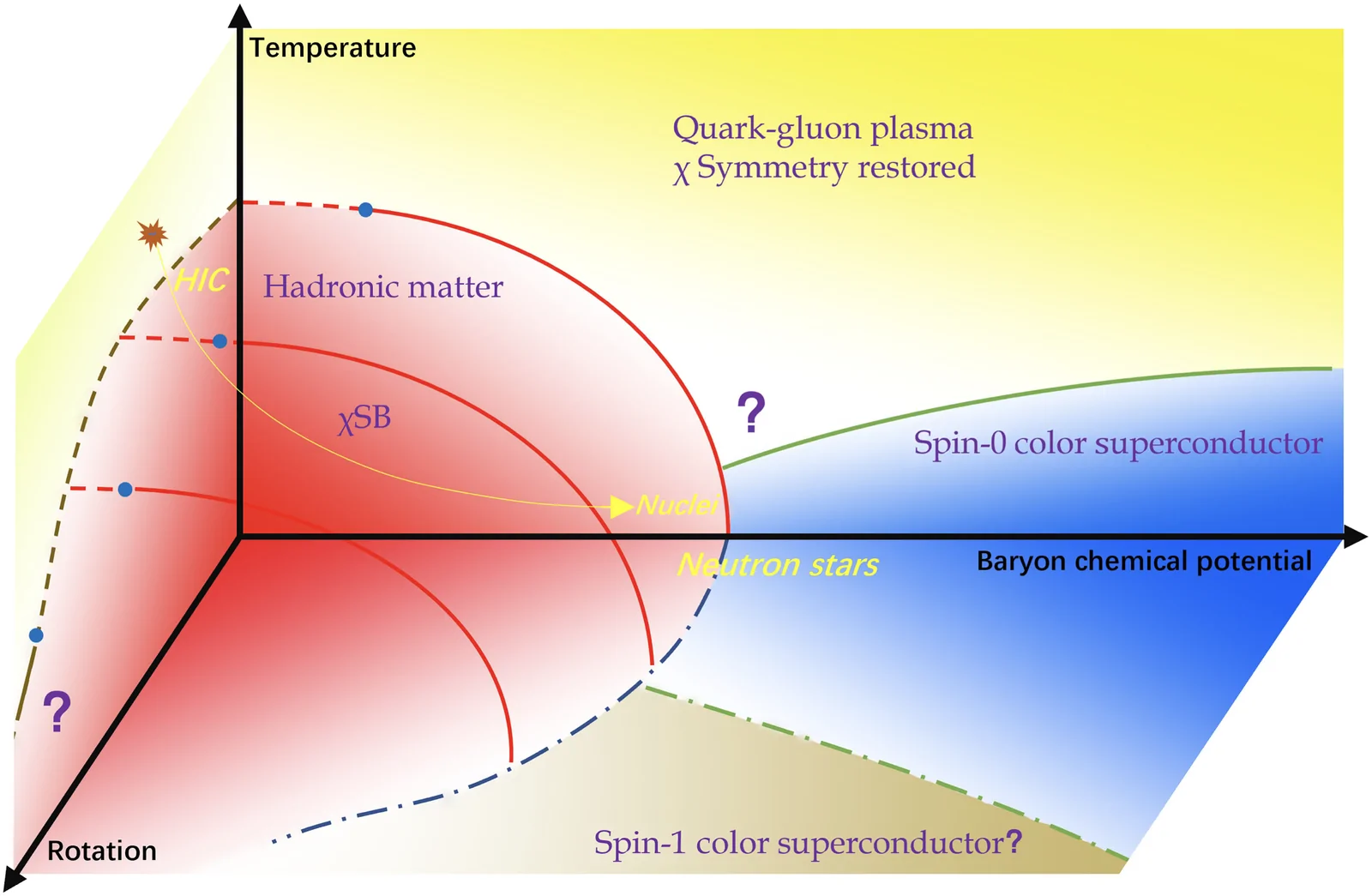
Rotation opens a new dimension for the phase diagram of QCD matter. The interplay between rotation, temperature, and baryon chemical potential leads to complex phase structures and rich phase transitions, as illustrated here. The traditional phase diagram on the “temperature (*T*)” and “baryon chemical potential μ_*B*_” plane features three major regions: hadronic matter at low *T* and μ_*B*_, QGP with restored chiral symmetry at high *T*, and spin-0 color superconductor at high μ_*B*_. The boundaries between these phases are phase transition lines in the above plot. The rotation ω opens a third dimension of the phase diagram. With increasing ω, the phase transition lines start to shift in nontrivial ways and possible new phases such as spin-1 color superconductor could emerge. Understanding these structures is important for the study of heavy-ion collisions in laboratories and of neutron stars in the sky. Reprinted from (*93*) with permission from Elsevier.

Traditionally, physicists have explored equilibrium phases of matter under certain macroscopic conditions such as temperature and chemical potential, which are “Lagrange multipliers” of conserved quantities like energy and charge density. Angular momentum **J** adds an entirely new dimension to the phase diagram of QCD matter, with vorticity ω being the corresponding Lagrangian multiplier. This is illustrated in Fig. 4. It is natural to ask: How the phases of QCD matter could be influenced by the presence of global angular momentum or equivalently nonzero vorticity field? The answer requires a deeper understanding of the equilibrium spin dynamics under rotation.

One prominent phenomenon of QCD phase structure is the spontaneous breaking of chiral symmetry (“χ SB”) in QCD vacuum, which accounts for most of the mass in our visible universe. While broken at low temperature and baryon chemical potential, it is restored in the high-temperature or high-density phases, as illustrated in Fig. 4. An interesting question is the effect of rotation on the χ SB. To do that, one needs to examine the spin and orbital structures of the chiral condensate, which can be understood as pairing between quark and antiquark (in analogy to Cooper pairing in superconductivity) (*85*). In this pairing configuration, the quark and antiquark have total spin *S* = 1 (i.e., with their spins in parallel) and total OAM *L* = 1, although they add up to have total angular momentum *J* = 0 (i.e., with *L* and *S* in opposite direction). Global rotation, on the other hand, prefers to polarize both *L* and *S* along the global angular momentum. As a result, the quark-antiquark pairing will be penalized by rotation and the chiral condensate will be suppressed more and more with increasing rotation. The chiral transition temperature is also found to decrease with increasing rotation, as indicated in Fig. 4.

The rotational suppression of fermion pairing in scalar configuration (i.e., with zero total angular momentum) is a generic phenomenon (*86*). Not unexpectedly, it has been shown that the same mechanism also leads to a suppression of the standard diquark pairing (in the Lorentz-scalar and isospin-scalar channel) in the color superconducting phase of QCD that is supposed to occur at low temperature and high baryon density. It also leads to a suppression of the so-called pion superfluidity phase at large isospin asymmetry (*87*). Obviously, if the rotation suppresses configurations with zero angular momentum, then it should somehow enhance configurations with nonzero angular momentum. This is indeed shown to be the case, implying an enhancement of vector meson condensation at large isospin density as well as a potential spin-1 color superconducting pairing at large baryon chemical potential. It is tempting to ask what possible rotational effects could be beyond just the fermions (i.e., quarks and antiquarks). Gluons in QCD are spin-1 particles and should be sensitive to global rotation. An outstanding question concerns how the QCD confinement transition is affected by rotation (*88*, *89*). These highly nontrivial aspects of the phases of rotating QCD matter are under active investigation. Although we are not yet at a point of fully answering these questions, lattice QCD calculations are extended to account for rotation and hold the promise of offering ab initio insights (*90*–*92*). See (*93*) for a review and references therein.

### Spin transport phenomena

The presence of angular momentum also brings a new category of dynamical transport phenomena, featuring the interplay between spin and orbital motion in nonequilibrium QCD matter. How does an initial angular momentum get distributed across the whole system and divided between the spin and orbital components? What is the equilibration condition between spin and orbital components? What determines the correlations and fluctuations of spin and that of the angular momentum in general? These questions cry out for answers, and their resolutions warrant a much deeper understanding of QCD spin dynamics.

A natural starting point is to figure out the global equilibrium state at finite angular momentum. Clearly, such a state necessitates a nontrivial fluid velocity field and thus can no longer be homogeneous in space. That is, the system needs to develop a local fluid field $u_{\mu }\left(x\right)$ (akin to velocity field v(x) in a nonrelativistic fluid) to accommodate the global angular momentum. Analyses based on quantum field theory and spin density matrix (*94*, *95*) suggest that the so-called “thermal vorticity” plays the central role, as defined below

$$\varpi_{\mu \nu }=-\frac{1}{2}\left[\partial_{\mu }\left(u_{\nu }/T\right)-\partial_{\nu }\left(u_{\mu }/T\right)\right]$$ (37)

One may recognize the above as a relativistic analogy of the $\left(\frac{\omega }{T}\right)$ factor in Eq. 36.

It serves as an effective “chemical potential” that couples to the angular momentum tensor in the density operator $∼\text{exp}\left(\varpi_{\mu \nu }J^{\mu \nu }/2\right)$ to determine the equilibrium distribution. Clearly, it ensures a rotational polarization of spin in equilibrium matter.

**Fig. 5.**
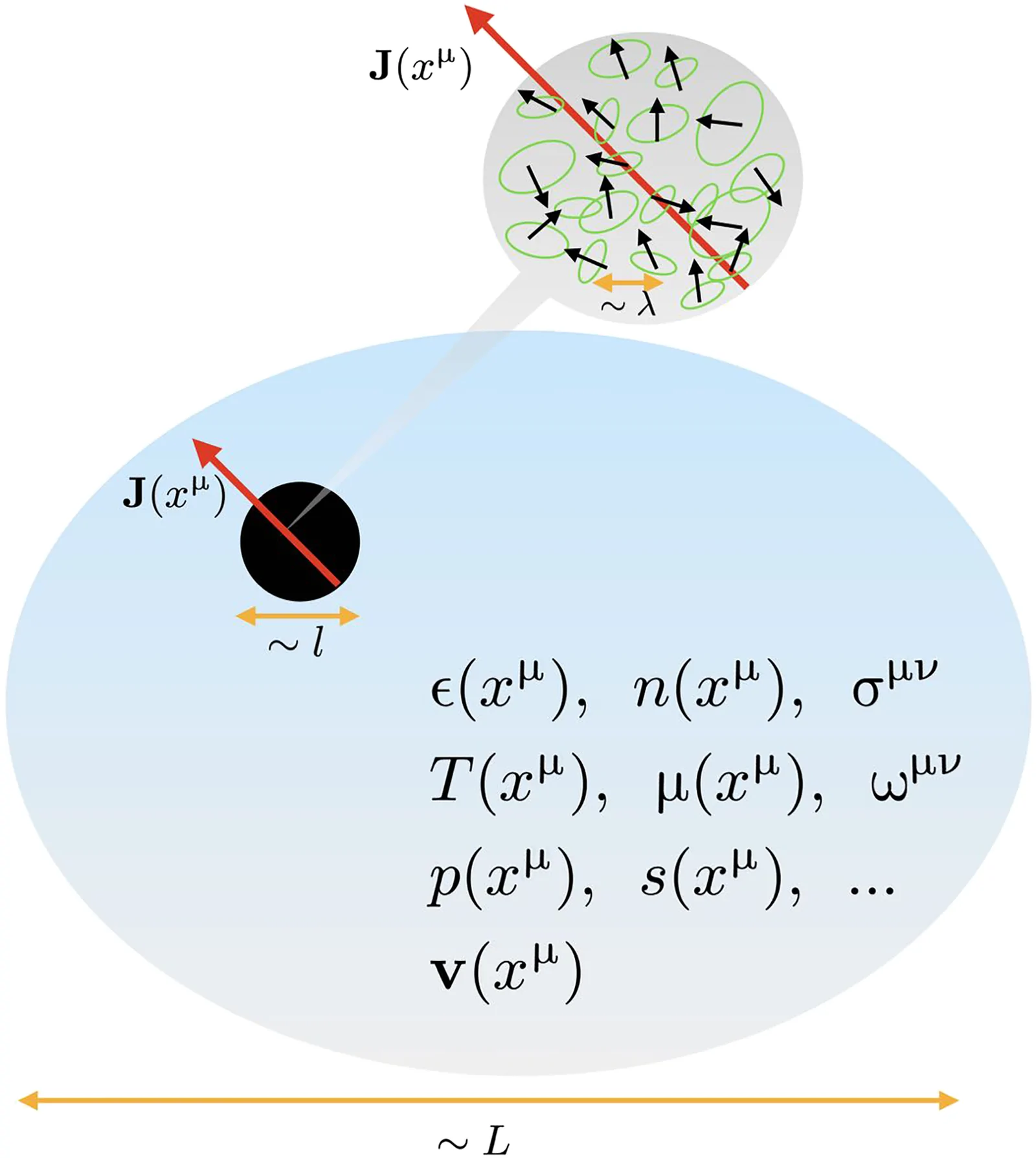
Spin hydrodynamics describes dynamical transport of angular momentum in strongly interacting matter. Reprinted from (*100*) with permission from Elsevier.

The next question is to consider the hydrodynamic regime, in which a system only stays in or close to local equilibrium and evolves toward global equilibrium. In the usual hydrodynamics framework, a many-body system is represented by hydrodynamic fields ϵ(x),n(x),T(x),μ(x),v,… representing local energy density, charge density, temperature, chemical potential, velocity, etc. They offer a coarse-grained description based on local fluid cells from summing and averaging over microscopic degrees of freedom, as depicted in Fig. 5. When angular momentum is included into consideration, there should then be a hydrodynamic field for local angular momentum tensor $J^{\mu \nu \rho }\left(x\right)$ as well as a corresponding “chemical potential” $\omega_{\mu \nu }\left(x\right)$. The usual hydrodynamic equations used to describe the evolution of these fields are based on conservation laws for energy-momentum tensor $T^{\mu \nu }\left(x\right)$ and charge currents $j^{\mu }\left(x\right)$ at the macroscopic scale. Let us now include an equation for the conservation law of angular momentum

$$\partial_{\mu }J^{\mu \nu \rho }\left(x\right)=0,\;J^{\mu \nu \rho }\left(x\right)=\left(x^{\nu }T^{\mu \rho }-x^{\rho }T^{\mu \nu }\right)+\Sigma^{\mu \nu \rho }$$ (38)

One may notice the decomposition of $J^{\mu \nu \rho }\left(x\right)$ into an orbital motion component and a spin current component $\Sigma^{\mu \nu \rho }$. While these are hydrodynamic fields defined at a certain macroscopic scale, they suffer an ambiguity (pertaining to the choice of pseudo-gauge) in such decomposition that is similar to their microscopic counterpart in quantum field operators, as already discussed in the nucleon spin case (*96*). Questions on whether a specific choice is more physical in connection with measurements in the context of spin hydrodynamics are still under intensive investigations; see, e.g., (*97*–*99*).

A core task of spin hydrodynamics is to construct the constitutive relation for the spin current $\Sigma^{\mu \nu \rho }$ through a systematic gradient expansion. In the ideal hydrodynamic limit with perfect local equilibrium, the spin current is simply given by $u^{\mu }\sigma^{\nu \rho }$ with $u^{\mu }$ the fluid four-velocity and $\sigma^{\nu \rho }$ the spin density in local rest frame. Here, spin density $\sigma^{\nu \rho }$ and spin chemical potential $\omega_{\nu \rho }$ are conjugate thermodynamic quantities just like charge density *n* and chemical potential μ. Beyond the ideal limit, derivative terms due to dissipative transport of angular momentum need to be included

$$\Sigma^{\mu \nu \rho }=u^{\mu }\sigma^{\nu \rho }+\tilde{\Sigma }{\left[u^{\kappa },\partial^{\lambda }\left(\omega^{\sigma \xi }/T\right)\right]}^{\mu \nu \rho }+\hat{O}\left(\partial \partial \right)$$ (39)

In the above, the first viscous term $\tilde{\Sigma }$ (i.e., Navier-Stokes term for spin) consists of various combinations of fields $u^{\kappa }\left(x\right)$ and $\partial^{\lambda }\left(\omega^{\sigma \xi }/T\right)$ with the proper overall Lorentz structure.

Their forms are completely fixed by the second law of thermodynamics, which requires the system entropy to never decrease. Much progress has been made recently in constructing such a spin hydrodynamic framework (*97*, *100*–*107*). Its application to phenomenological modeling of spin transport in heavy-ion collisions is also under exploration.

A more microscopic approach to study spin transport is provided by quantum kinetic equations. A natural framework to derive these equations is the Wigner function formalism. Using a semiclassical expansion in ℏ, one can systematically obtain transport equations for phase-space distributions order by order. While the zeroth order gives classical Boltzmann transport, the ℏ order leads to a description of the spin degree of freedom and its evolution. Collision terms accounting for spin-dependent scatterings can also be included. This theoretical framework proves to be a powerful tool for the development of spin hydrodynamics. It has also found various applications in the phenomenological studies of spin transport effects observed in heavy-ion collisions. One may realize the limitation in the applicability of kinetic equations for realistic systems. To that end, a strong-coupling method based on holographic models of QCD offers a complementary approach for understanding spin transport in QCD matter under rotation.

### Spin transport measurements in heavy-ion collisions

In relativistic heavy-ion collisions, two atomic nuclei are accelerated to high energy and collide to create a new form of QCD matter with extreme temperatures. This hot matter then experiences an explosive evolution that is well described by relativistic viscous hydrodynamics. For decades, research in this field focused on the transport of energy and momentum in this highly dynamical fluid, while the angular momentum associated with this colliding system was largely ignored. A back-of-the-envelope estimate suggests its existence in a noncentral collision

$$J_{y}=\frac{\mathrm{Ab}\sqrt{s_{\text{NN}}-{\left(2m_{N}\right)}^{2}}}{2}$$ (40)

where $J_{y}$ is the angular momentum component perpendicular to the reaction plane, *A* is the number of nucleons, *b* is the impact parameter (i.e., distance between the center of the two nuclei), $\sqrt{s_{\text{NN}}}$ is the collisional beam energy, and $m_{N}$ is the nucleon mass. For typical collisions at RHIC and LHC energies, it can easily reach $10^{\left(5∼6\right)}\;\hbar$, which is substantial. The real question is: Are there any observable consequences of this large angular momentum?

**Fig. 6.**
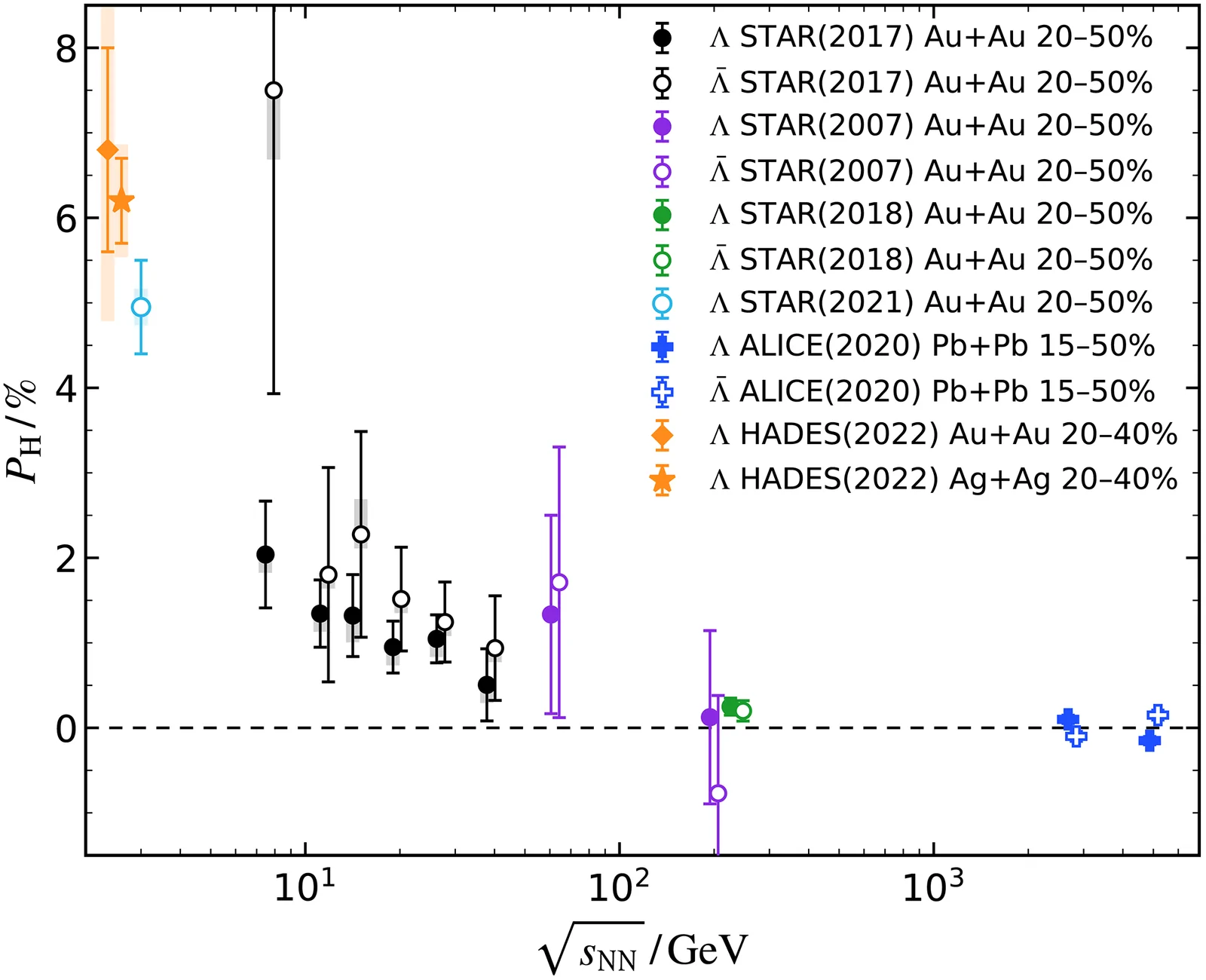
Global spin polarization was found in heavy-ion collisions. Global spin polarization measurements of hyperons Λ and antihyperons $\bar{\Lambda }$ are shown for a broad range of collisional beam energy $\sqrt{s_{\text{NN}}}$, from $\hat{O}\left(1\right)$ to $\hat{O}\left(1000\right)$ GeV. These include results from STAR, ALICE, and HADES experiments (*17*, *162*–*167*). The signal strength is found to rapidly increase with decreasing beam energy, reaching a level of several percent at low beam energy.

It was first proposed by Liang and Wang around 2004 that such an angular momentum could turn into measurable signals via global spin polarization of hyperons and spin alignment of vector mesons (*108*, *109*). A fluid dynamical scenario was proposed by Becattini *et al.* around 2007 and subsequently developed by many into today’s standard paradigm (*94*, *95*). Namely, this angular momentum in Eq. 40 leads to a strong fluid vorticity field that evolves together with the system and induces spin polarization of final-state hadrons upon hydrodynamic freeze-out. Initial effort by the STAR Collaboration started almost immediately to measure this signal in top energy collisions at RHIC, yet without success. A crucial insight about the beam energy dependence of the vorticity field was found later (*110*, *111*), predicting its strong increase with decreasing beam energy, in a trend opposite to the total angular momentum itself. Thanks to high-statistics datasets from the RHIC BES experiments (*112*) with precision tracking and complete kinematic coverage at collider energies, global hyperon spin polarization was found and reported in a 2017 *Nature* publication by the STAR Collaboration (*17*). A signal at a few percent level was unambiguously observed, in the low–beam energy region as expected: See Fig. 6. Following that, a whole slew of related measurements has begun a new era of quantitative characterization of the vorticity structures in a subatomic fluid of QCD matter. The injection of this new degree of freedom has since presented an enormous opportunity to generate new insights into the spin dynamics of QCD matter.

To quantitatively connect the measured spin polarization signal with the fluid vorticity, one can examine the mean spin vector $S^{\mu }\left(p\right)$ of hadrons produced upon freeze-out. For a spin $\frac{1}{2}$ fermion with momentum *p* and mass *m* at local equilibrium (*95*), this is given by

$$S^{\mu }\left(p\right)=-\frac{1}{8m}\epsilon^{\mu \nu \rho \sigma }p_{\sigma }\frac{\int_{\Sigma }d\Sigma \cdot p\varpi_{\nu \rho }n_{F}\left(1-n_{F}\right)}{\int_{\Sigma }d\Sigma \cdot {\mathrm{pn}}_{F}}$$ (41)

where $n_{F}$ is the covariant Fermi-Dirac distribution function and Σ represents the freeze-out hypersurface. The thermal vorticity $\varpi_{\nu \rho }$ introduced in (*38*) plays a central role in the above equation, which is the freeze-out scheme for spin degrees of freedom in analogy to the well-known Cooper-Frye freeze-out scheme for particle production. It should be emphasized that this result essentially comes from the rotational polarization of spin, as illustrated in Eq. 36 and discussed in the “Rotational polarization of spin” section. The above equation can be interpreted as the leading-order difference between the Boltzmann factors of spin up or down states, $∼\left(e^{\varpi \cdot \left(+1/2\right)}-e^{\varpi \cdot \left(-1/2\right)}\right)∼\varpi$, to be summed over all particles produced at freeze-out. Equation 41 also provides a basis for the fluid dynamical scenario of spin polarization: Initial angular momentum $J_{y}$↔ nonzero rotational vorticity 〈ϖ〉↔ spin polarization $P_{H}$ of produced particles along the global angular momentum direction. The data shown in Fig. 6 were quantitatively understood in a robust way by various bulk evolution models of heavy-ion collisions, thus providing a clear indication that we have at hand a paradigm to interpret such signals in terms of the rotational polarization of spin, as predicted in the “Rotational polarization of spin” section. For a detailed review, see, e.g., (*113*). On the basis of the 2017 STAR measurements, an average vorticity was estimated to be $\left(9\pm 1\right)\times 10^{21}\;s^{-1}$, crowning the subatomic swirls created in heavy-ion collisions with the title of “the most vortical fluid.”

The global polarization in (*42*) is an integral effect related to the average vorticity field. More differential measurements have been developed to unravel the local polarization features, such as longitudinal and transverse spin polarization and their dependence on momentum and azimuthal angle. Such detailed information allows ongoing efforts toward fully mapping out the rich and complex vorticity structures in this subatomic fluid, and brings unexpected new insights. For example, while the global polarization signal can be straightforwardly and quantitatively described by various hydrodynamic or transport model simulations, the explanation of local polarization patterns appears to necessitate the inclusion of a previously unknown contribution that comes from the shear stress tensor of the fluid field and is previously overlooked (*114*–*117*). Comparisons between models and data also allude to their nontrivial sensitivity to the three-dimensional initial conditions of fluid evolution (*118*), potentially allowing spin measurements to serve as diagnostic probes. There has also been a highly creative idea to create and detect local vorticity configurations akin to “smoke rings” in, e.g., proton-nucleus collisions (*119*).

Beyond Λ and $\bar{\Lambda }$ hyperons, experimental measurements and theoretical studies of spin transport in heavy-ion collisions have been extended to an abundance of particle species, from spin-$\frac{1}{2}$ multistrangeness hyperons and various spin-1 vector mesons to spin-$\frac{3}{2}$ hadrons and even light nuclei. A notable challenge is presented by the global spin alignment patterns of vector mesons, which have so far been measured at RHIC and the LHC for $K^{*0}$, ϕ, $D^{*+},J/\psi$, and even ϒ states, over a broad range of beam energies and different kinematic regimes (*120*–*123*). The relevant observable is $\rho_{00}$, the 00-component of the spin density matrix $\rho_{\mathrm{ij}}$ for describing spin states of a spin-1 particle. If induced by the fluid vorticity, it deviates from an isotropic baseline value of 1/3 according to $\rho_{00}\approx \frac{1}{3}-\frac{4}{8}{\langle \varpi \rangle }^{2}$. While this observable is found to qualitatively align with the fluid vorticity interpretation, its magnitude markedly exceeds the level implied by the hyperon global spin polarization and thus strongly indicates alternative sources of contribution. Elsewhere, the $\rho_{00}$ is also found to be around 1/3 for certain particles in certain regions and to even go above 1/3 in some other cases. As of now, there is no simple and coherent understanding of these data.

Opening a new chapter of this ongoing story, the STAR experiment recently reported in (*124*) the first evidence of spin correlations in $\Lambda \bar{\Lambda }$ hyperon pairs produced in proton-proton collisions at RHIC. The results reveal a relative polarization signal that links the virtual spin-correlated quark pairs from the QCD vacuum to their final-state hadron counterparts. It is of great interest to investigate how this correlation evolves from proton-proton to nucleus-nucleus collisions. Future measurements along this line could provide a new pathway to explore the interplay between spin transport in hot QCD matter and the hadronization mechanism.

### Outlook

To wrap it up, a new field of studying spin dynamics in strongly interacting matter has emerged from recent experimental discoveries and theoretical developments. It features a robust foundation of robust physics underpinning and rich phenomena both in and out of equilibrium, along with intriguing puzzles, surprising twists, and interdisciplinary connections. The present status of the field is inviting: There is simply more to come. The interested readers are referred to a 2021 book entitled “*Strongly Interacting Matter under Rotation*” (*113*) (where each chapter presents an in-depth overview on a specific topic) as well as to more recent reviews (*125*–*127*) and references therein.

So, where are we headed next? On the experimental side, it is almost certain that the already comprehensive datasets will continue to expand and improve, culminating in a wealth of high-precision spin transport measurements across particle species, colliding systems, beam energies, and kinematic regions. The challenge will fall more on the theoretical side, namely, how to navigate from signal to physics and achieve a synergy of understanding. On the flip side, there are ample opportunities. One expects to see future progress in a number of areas, as discussed in a selected list below:

1) Quantifying the early stage dynamics, including the initial conditions of angular momentum, their relations with other phenomena (such as glasma fields or baryon stopping), and the pre-hydro spin transport.

2) Completing the theoretical construction of spin hydrodynamic framework, and developing full-fledged phenomenological simulations that describe the dynamical evolution of vorticity fields and provide a unified explanation of global and local spin polarization patterns.

3) Using advanced tools like Bayesian inference and machine learning to extract key transport coefficients of spin transport from experimental data, and deciphering their microscopic interpretations in terms of the underlying QCD spin dynamics.

4) Identifying key physical mechanisms of the observed spin alignment, and achieving a coherent understanding of its variation with different measurement settings as well as its relation with spin polarization observables.

5) Understanding the sources of spin correlations and their roles in the formation of hadrons from the QGP.

6) Characterizing equilibrium properties of QCD matter with angular momentum, such as equation of state and phase structures, with both effective field theories and lattice QCD simulations.

## ANOMALOUS TRANSPORT AND CME

Spin, helicity, and chirality are closely intertwined concepts. From the viewpoint of Lorentz symmetry, chirality serves as a fundamental concept in defining spinor fields, as it labels the finite-dimensional “left” and “right” irreducible representations of the complexified Lie algebra of the Lorentz group: $\mathrm{SO}{\left(3,1\right)}_{C}=\mathrm{SU}{\left(2\right)}_{L}⊕\mathrm{SU}{\left(2\right)}_{R}$. Chirality also plays a central role in phenomena pertaining to quantum anomalies and gauge field topology. However, unlike spin or helicity, chirality is difficult to access directly in experiments. Recently, nontrivial ways of manifesting chirality via measurable effects in macroscopic physical systems have been found, as we are going to describe in this section.

### From quantum anomalies to transport

Quantum anomalies occupy a special place in quantum field theory. They represent a subtle interplay between symmetry and quantization: A symmetry of the classical action fails to survive at the quantum level because of ultraviolet regularization. Among the most profound examples is the chiral anomaly, which ties the nonconservation of chiral charge to topology and to gauge fields (*25*, *26*). In relativistic many-body systems, the anomaly has remarkable macroscopic consequences: It induces nondissipative transport currents along magnetic fields and vorticity. These phenomena collectively form the subject of anomalous transport.

Perhaps the most prominent example is the CME (*128*–*130*): In a system with a chiral imbalance, a magnetic field generates an electric current parallel to itself; for reviews, see (*131*–*134*)

$$J=\frac{e^{2}}{2\pi^{2}}\;\mu_{5}\;B$$ (42)

where $\mu_{5}$ is the chiral (axial) chemical potential and B is the magnetic field. This current is even under time-reversal and thus dissipationless; its magnitude is completely fixed by the anomaly coefficient. Equation 42 implies that topology and quantum anomalies affect macroscopic transport.

The CME was first discussed in the context of QCD and heavy-ion collisions and subsequently generalized to condensed matter systems such as Dirac and Weyl semimetals. Over the past decade, anomalous transport has become a unifying theme connecting spin physics, topology, relativistic hydrodynamics, and quantum information.

In this section, we provide a pedagogical introduction to anomalous transport, focusing on the CME. We proceed from the chiral anomaly to macroscopic transport, and review its experimental status in heavy-ion collisions, including recent results from the BES at RHIC.

### Chiral anomaly: From spin to topology

Consider a massless Dirac fermion coupled to an electromagnetic field

$$L=\bar{\psi }i\gamma^{\mu }D_{\mu }\psi ,\;D_{\mu }=\partial_{\mu }+{\mathrm{ieA}}_{\mu }$$ (43)

Classically, in the massless limit, the theory has a conserved vector current $J^{\mu }=\bar{\psi }\gamma^{\mu }\psi$ and a conserved axial current $J_{5}^{\mu }=\bar{\psi }\gamma^{\mu }\gamma^{5}\psi$. However, quantum mechanically

$$\partial_{\mu }J_{5}^{\mu }=\frac{e^{2}}{16\pi^{2}}F_{\mu \nu }{\tilde{F}}^{\mu \nu }=\frac{e^{2}}{2\pi^{2}}\;E\cdot B$$ (44)

where ${\tilde{F}}^{\mu \nu }=\frac{1}{2}\epsilon^{\mu \nu \alpha \beta }F_{\alpha \beta }$.

Equation 44 is the chiral anomaly, often also referred to as the Adler-Bell-Jackiw anomaly (*25*, *26*). It links the nonconservation of axial charge

$$Q_{5}=\int d^{3}x\;J_{5}^{0}$$ (45)

to the topological density $F\tilde{F}$ of the gauge field. In QCD, the anomaly relates the axial charge of quarks (with $N_{f}$ flavors) to the topology of gluons

$$\partial_{\mu }J_{5}^{\mu }=\frac{g^{2}N_{f}}{16\pi^{2}}G_{\mu \nu }^{a}{\tilde{G}}^{a\;\mu \nu }$$ (46)

where $G_{\mu \nu }^{a}$ and ${\tilde{G}}^{a\;\mu \nu }$ are gluon field strength tensor and its dual tensor. By integrating the above equation over spacetime, one arrives at the following relation

$$Q_{5}=2N_{f}Q_{w},\;Q_{w}\equiv \frac{g^{2}}{32\pi^{2}}\int d^{4}x\;G_{\mu \nu }^{a}{\tilde{G}}^{a\;\mu \nu }$$ (47)

$Q_{w}$ is the topological winding number associated with gluonic field configurations known as sphalerons and instantons, which are transitions between topologically distinct sectors of gauge field vacua. The above relation, deeply connected with the celebrated Atiyah-Singer index theorem in mathematics, makes a powerful statement: fluctuations of gauge field topology generate chirality.

The chiral anomaly can be understood physically in terms of spin and spectral flow (*135*). In a magnetic field, fermions occupy Landau levels. The lowest Landau level (LLL) is special: It is effectively (1 + 1) dimensional and has its spin locked to the direction of motion. Right- and left-handed fermions propagate along or against the magnetic field. An electric field parallel to B causes spectral flow between negative and positive energy states, creating an imbalance in chirality. This intuitive picture directly leads to Eq. 44.

Consider a constant magnetic field $B=B\hat{z}$. The spectrum of massless Dirac fermions is

$$E_{n,p_{z}}^{2}=p_{z}^{2}+2|\mathrm{eB}|n,\;n=0,1,2,\ldots$$ (48)

Note that spin plays a crucial role in the structure of the above Landau levels through magnetic moment interaction with the B field. The lowest Landau level (n=0) is chiral

$$E_{0,p_{z}}=\pm p_{z}$$ (49)

with the sign determined by chirality, while the excited levels are degenerate in spin.

Assume different Fermi momenta for right- and left-handed fermions

$$p_{F}^{R,L}=\mu_{R,L}$$ (50)

The density of states per unit transverse area in the LLL is

$$\frac{|\mathrm{eB}|}{2\pi }$$ (51)

The electric current along *z* is

$$J_{z}=\frac{e|\mathrm{eB}|}{2\pi }\left(\int_{0}^{\mu_{R}}\frac{{\mathrm{dp}}_{z}}{2\pi }-\int_{0}^{\mu_{L}}\frac{{\mathrm{dp}}_{z}}{2\pi }\right)$$ (52)

$$=\frac{e^{2}B}{4\pi^{2}}\left(\mu_{R}-\mu_{L}\right)=\frac{e^{2}}{2\pi^{2}}\mu_{5}B$$ (53)

where $\mu_{5}=\left(\mu_{R}-\mu_{L}\right)/2$ quantifies the chirality imbalance. This reproduces Eq. 42.

Several features of this result deserve emphasis: (i) only the lowest Landau level contributes; (ii) the result is independent of temperature; (iii) the coefficient is protected by the anomaly and not renormalized by interactions (however, the magnetic field itself can be modified in the medium). The CME is therefore topological and universal.

### CME as a nonequilibrium phenomenon

The CME Eq. 42 relates a vector current to a magnetic field in the presence of a chiral chemical potential $\mu_{5}\equiv \left(\mu_{R}-\mu_{L}\right)/2$. Although Eq. 42 resembles a static linear-response relation, the CME is intrinsically a nonequilibrium transport phenomenon. Its very existence relies on the dynamical generation and relaxation of axial charge through the chiral anomaly.

Consider a system described by a stationary density matrix

$$\rho =\frac{1}{Z}\text{exp}\left[-\beta \left(H-\mu Q-\mu_{5}Q_{5}\right)\right]$$ (54)

with vector charge *Q* and axial charge $Q_{5}$. If $\mu_{5}$ were a genuine thermodynamic chemical potential corresponding to a conserved charge, then Eq. 42 would imply a persistent vector current in equilibrium. However, such a current would correspond to a net transport of electric charge in the ground state and is forbidden in normal (nonsuperconducting) phases by general principles such as Bloch’s theorem and gauge invariance. A careful treatment of the static Kubo formula confirms that the equilibrium CME current vanishes once the correct order of limits is taken in the infrared.

The key observation is that axial charge is not conserved. In relativistic quantum field theory it obeys the anomaly Eq. 44. Even in the chiral limit m→0, the axial current is not conserved in backgrounds with E⋅B≠0 or, in non-Abelian gauge theory, in topologically nontrivial gluon configurations. Consequently, $Q_{5}$ cannot be defined as a truly conserved charge in thermodynamics. Any initially prepared $\mu_{5}$ relaxes on a characteristic time scale $\tau_{5}$ set by chirality-changing processes.

Thus, the CME current in Eq. 42 must be interpreted as a transport current sustained by a time-dependent chiral imbalance. In strict global equilibrium, $\mu_{5}\to 0$ and the CME vanishes, just as in the early proposal by Vilenkin (*136*), where the chiral chemical potential was induced by parity violation in weak interactions.

In the QGP produced in heavy-ion collisions at RHIC and the LHC, a chiral chemical potential is not imposed externally; it is generated dynamically by topological fluctuations of the gluon fields. At finite temperature, non-Abelian gauge theory admits real-time transitions between gauge configurations with different Chern-Simons number $N_{\text{CS}}$. These transitions are mediated by sphalerons—unstable classical field configurations representing the top of the potential barrier between neighboring topological sectors. These transitions are accompanied by a change $\Delta N_{\text{CS}}$ which equals the topological charge $Q_{w}$ of sphalerons. They induce axial charge production via the anomaly as in Eq. 47.

In the deconfined phase, the sphaleron transition rate scales parametrically as (*137*)

$$\Gamma_{\text{sph}}∼\alpha_{s}^{5}\;T^{4}$$ (55)

leading to stochastic fluctuations of axial charge density. Event by event, domains with nonzero $n_{5}$ (and hence $\mu_{5}$) are created. Because these transitions are random in sign, $\langle \mu_{5}\rangle =0$ over many events, but $|\mu_{5}|$ is nonzero in individual events.

In noncentral heavy-ion collisions, extremely strong magnetic fields

$$\mathrm{eB}∼m_{\pi }^{2}$$ (56)

are generated by the fast-moving ions (*129*, *138*). The simultaneous presence of strong B and fluctuating $\mu_{5}$ produces a charge separation along the magnetic field direction via the CME. Both the magnetic field and the axial charge are time dependent; therefore, the phenomenon is inherently far from equilibrium and exists only during the short lifetime of the plasma.

The CME has been observed experimentally (*19*, *20*) in condensed-matter systems with chiral quasiparticles, such as Weyl and Dirac semimetals. Near Weyl nodes, the low-energy excitations behave as massless chiral fermions, and the axial anomaly manifests itself in momentum space. In these materials, the axial charge density satisfies

$$\partial_{t}n_{5}=\frac{e^{2}}{2\pi^{2}}E\cdot B-\frac{n_{5}}{\tau_{v}}$$ (57)

where $\tau_{v}$ is the intervalley scattering time that relaxes chirality. Parallel electric and magnetic fields pump chiral charge between Weyl nodes of opposite chirality. In steady state

$$n_{5}=\frac{e^{2}\tau_{v}}{2\pi^{2}}E\cdot B$$ (58)

Substituting the corresponding chiral chemical potential into Eq. 42 yields a current proportional to $B^{2}$, leading to negative longitudinal magnetoresistance (*139*)—a characteristic experimental signature of the anomaly.

Chiral imbalance can also be generated optically. Circularly polarized light selectively excites carriers in one Weyl node more efficiently than in the other, producing a transient $\mu_{5}$ even without static E⋅B. Once pumping ceases, intervalley scattering relaxes $n_{5}$, again underscoring that the CME is sustained only in nonequilibrium conditions.

### Anomalous hydrodynamics

In relativistic hydrodynamics, the conserved currents are expanded in gradients of fluid velocity $u^{\mu }$, temperature *T*, and chemical potentials. In addition to ordinary dissipative terms, anomalies generate nondissipative contributions.

For a single *U* (*1*) current in the presence of a chiral chemical potential, the constitutive relation reads

$$J^{\mu }={\mathrm{nu}}^{\mu }+\sigma_{B}B^{\mu }+\sigma_{\omega }\omega^{\mu }+\ldots$$ (59)

where

$$B^{\mu }=\frac{1}{2}\epsilon^{\mu \nu \alpha \beta }u_{\nu }F_{\alpha \beta },\;\omega^{\mu }=\frac{1}{2}\epsilon^{\mu \nu \alpha \beta }u_{\nu }\partial_{\alpha }u_{\beta }$$ (60)

The transport coefficients are fixed by anomalies

$$\sigma_{B}=\frac{e^{2}}{2\pi^{2}}\mu_{5},\;\sigma_{\omega }=\frac{e{\mu \mu }_{5}}{\pi^{2}}+\frac{T^{2}}{6}$$ (61)

The second term corresponds to the chiral vortical effect. Hydrodynamic consistency together with the anomaly uniquely determines these coefficients (*82*).

### CME in heavy ion collisions

In heavy-ion collisions, noncentral collisions generate extremely strong magnetic fields ($|\mathrm{eB}|∼m_{\pi }^{2}$ or larger) at early times. At the same time, topological gluonic transitions can produce local fluctuations of axial charge. According to Eq. 42, this leads to an electric current along B, separating positive and negative charges across the reaction plane.

Because the sign of topological charge fluctuates event by event, the average current vanishes, but charge-dependent correlations remain. Experimentally, one measures azimuthal correlations such as (*140*)

$$\gamma_{\alpha \beta }\equiv \langle \text{cos}\left(\phi_{\alpha }+\phi_{\beta }-2\Psi_{\text{RP}}\right)\rangle$$ (62)

in which $\phi_{\alpha }$ and $\phi_{\beta }$ are the azimuthal angles for a pair of produced charged hadrons, $\Psi_{\text{RP}}$ is the azimuthal angle of the collisional reaction plane, the indices α,β=± label the sign (positive or negative) of a hadron’s charge, and 〈…〉 denotes an average over all events. Such correlations are sensitive to charge separation relative to the reaction plane angle $\Psi_{\text{RP}}$. However, other conventional correlations could also contribute to these correlations. A major challenge is to disentangle the CME signal from background correlations driven by elliptic flow, as we will now discuss in detail.

A CME-induced electric current along the magnetic field, which is approximately perpendicular to the reaction plane in noncentral collisions, generates a charge separation that leads to a difference between the opposite-sign pair correlation and the same-sign pair correlation, quantified by

$$\Delta \gamma \equiv \gamma_{+-}-\frac{1}{2}\left(\gamma_{++}+\gamma_{--}\right)$$ (63)

In the above, $\gamma_{++}$ and $\gamma_{--}$ measure azimuthal correlations between same-sign hadron pairs, while $\gamma_{+-}$ quantifies those between opposite-sign hadron pairs. The first measurements by the STAR Collaboration in 2009 appeared in line with CME expectations (*141*, *142*). It was, however, quickly realized that the Δγ correlator is parity even and therefore receives substantial contributions from background correlations unrelated to the CME. The dominant background arises from local charge conservation coupled to elliptic flow $v_{2}$, which produces charge-dependent correlations aligned with the event plane (*143*). Additional sources include resonance decays, momentum conservation, cluster correlations, and other collective effects (*144*–*146*). Since many of these backgrounds scale approximately with $v_{2}$, a central experimental challenge is to disentangle a possible CME contribution, expected to scale with the magnetic field strength, from flow-driven backgrounds (*147*).

To address this, a variety of strategies have been developed: comparison between isobar systems with similar geometry but different magnetic fields, event-shape engineering to vary $v_{2}$ at fixed centrality, measurements relative to the spectator plane, and beam-energy scans that exploit the nontrivial energy dependence of the magnetic field lifetime and medium properties. See detailed discussions in, e.g., (*131*, *134*, *147*–*151*). The quantitative interpretation of Δγ therefore requires careful modeling of both anomalous transport and background correlations, making the separation of CME signals from conventional QCD dynamics one of the central challenges in this field. A state-of-the-art simulation framework, the “event-by-event anomalous-viscous fluid dynamics” (EBE-AVFD), was developed to address such a critical need (*152*–*154*). It provides a powerful theoretical tool that has now been widely used to develop new analysis methods, to quantify backgrounds and signal response, as well as to help interpret experimental measurements (*149*).

The analyses combining isobar collisions (Ru4496+Ru4496 and Zr4096+Zr4096) and BES data provide important constraints on the CME fraction in the observed correlations. The isobar program exploits the difference in nuclear charge (and hence magnetic field) while keeping geometry similar (*148*). The initial blind analysis by STAR Collaboration was inconclusive because of a small but non-negligible difference in the measured event multiplicity and elliptic flow between the isobar pair (*155*). By accounting for this subtle difference, a theoretical analysis based on the EBE-AVFD framework was able to identify a finite CME signal contribution of about (6.8 ± 2.6)% in the isobar data (*156*). Subsequent postblind analyses from STAR (*157*, *158*) extracted an upper limit of 10% at 95% confidence level for the CME contribution, consistent with the theoretical result.

Recent high-statistics measurements by the STAR Collaboration in the RHIC BES program (*112*) have provided intriguing indications of the CME in heavy-ion collisions (*18*, *159*). In Au + Au collisions over a broad range of center-of-mass energies ($\sqrt{s_{\text{NN}}}=7.7-200\;\text{GeV}$), charge separation signals perpendicular to the spectator plane have been extracted using a newly developed event shape selection technique that effectively suppresses flow-induced backgrounds. After extrapolation toward the zero–elliptic flow limit, a residual charge separation consistent with CME expectations persists in mid-central collisions at $\sqrt{s_{\text{NN}}}=11.5$, 14.6, and 19.6 GeV with statistical significances of approximately 2.6σ, 3.1σ, and 3.3σ, respectively. These signals are statistically limited or consistent with zero at both lower and higher beam energies, including the top RHIC energy of 200 GeV, where background effects dominate. When data between $\sqrt{s_{\text{NN}}}=10$ and 20 GeV are combined, the overall significance of a nonzero charge separation increases to more than 5σ, suggesting the possible emergence of CME-related effects in an energy window where magnetic field dynamics and chiral symmetry restoration may be favorable. These findings, reported in (*18*, *159*), represent some of the most compelling experimental evidence to date for anomaly-induced transport phenomena in QCD matter produced in relativistic heavy-ion collisions. A recent theoretical analysis combining EBE-AVFD simulations with data-driven machine learning methodology is able to provide a quantitative interpretation of these measurements and to unambiguously extract a nonzero contribution from CME transport.

**Fig. 7.**
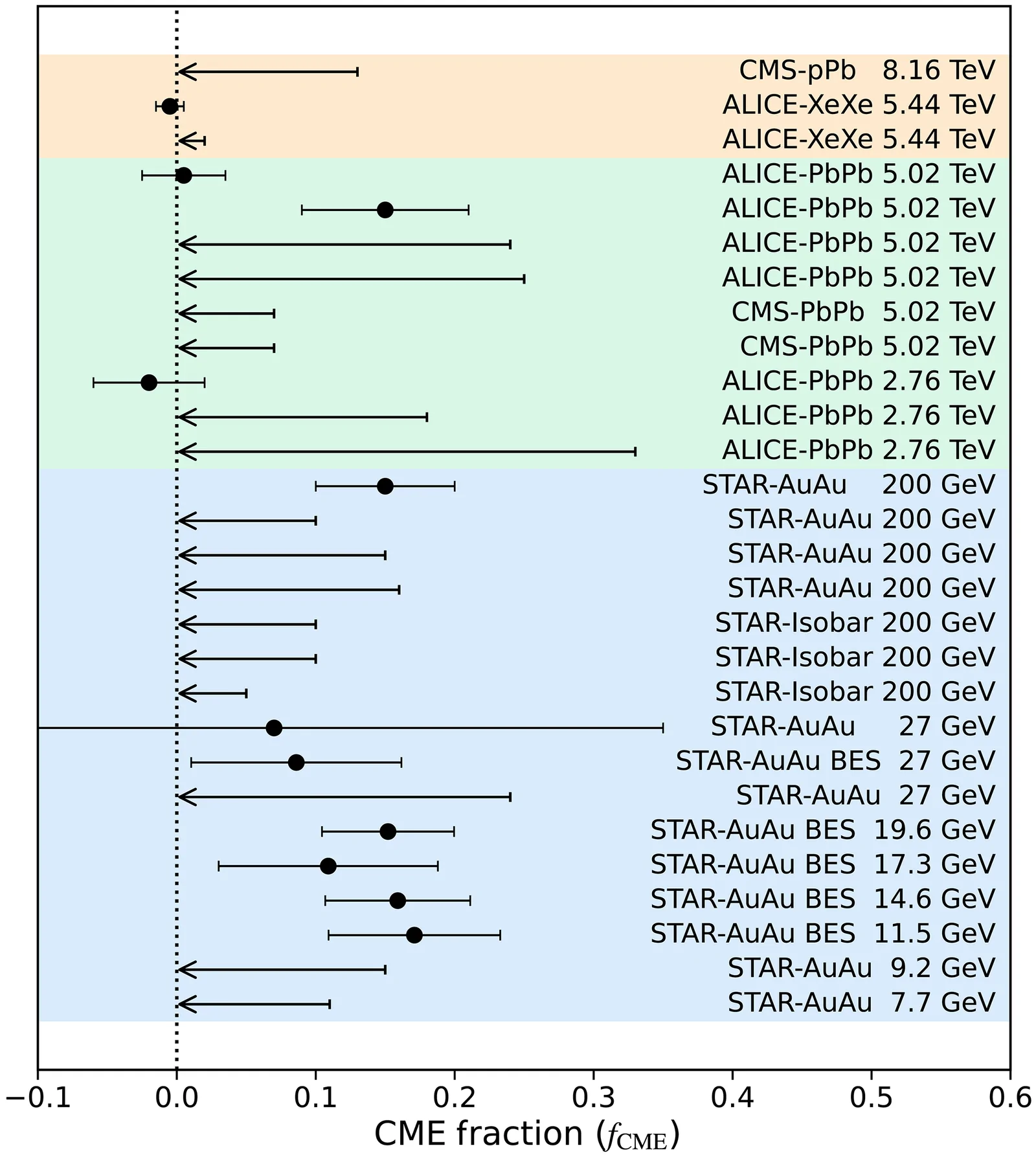
Potential signals of CME in heavy-ion collisions are extracted from various experiments. This plot compiles various measurements for the fraction of CME signal or its upper limit extracted from the azimuthal correlator Eq. 62, by STAR, ALICE, and CMS collaborations at RHIC and the LHC over a span of beam energy from 7.7 GeV to 8.16 TeV for a variety of colliding systems. Sources for these measurements can be found in (*18*, *131*, *155*, *157*–*159*, *168*–*175*).

Last, the current status of the experimental search is summarized in Fig. 7. The potential CME signal is quantified as CME fraction, *f*_CME_, defined as the fraction of the CME contribution in the measured total azimuthal correlation Δγ defined in Eq. 63.

### Why the CME may be enhanced at low energies

One of the intriguing features of the BES program is the possibility that the CME may become more prominent at lower center-of-mass energies. Several dynamical mechanisms suggest that the conditions required for anomalous transport—namely, a strong magnetic field and a sustained chiral imbalance—may be more favorable in this regime, as we shall discuss next.

#### Longer-lived magnetic field

In noncentral heavy-ion collisions, intense magnetic fields are generated primarily by the fast-moving spectator protons. At top RHIC and LHC energies, these spectators move ultrarelativistically and rapidly recede from the interaction region, leading to a very short-lived magnetic field pulse. Although the peak field strength can be extremely large, its duration is parametrically suppressed by Lorentz contraction and by the rapid separation of the spectator charges.

At lower beam energies, the spectators move more slowly and remain in the vicinity of the fireball for a longer time. As a result, the magnetic field decays more gradually. In addition, baryon stopping becomes substantial in this regime: A large fraction of the electric charge carried by the incoming nuclei is deposited in the interaction region. The presence of these stopped charges could increase the electric conductivity of the medium and enhance the electromagnetic response of the QGP. A conducting plasma supports induced currents that, via Faraday’s law, tend to sustain the magnetic field and slow its decay. The coupled Maxwell-hydrodynamic evolution therefore leads to a longer effective lifetime of the magnetic field in the medium rest frame. Since the CME current is proportional to **B**, an extended magnetic field lifetime increases the integrated charge separation signal.

#### Enhanced topological fluctuations

A second potential source of enhancement arises from the QCD phase structure. The BES program explores a region of the phase diagram at finite baryon chemical potential μ_*B*_, where a critical point associated with the chiral phase transition may exist. Near a critical point, long-wavelength fluctuations of the order parameter become large, and correlation lengths grow. Since topological charge fluctuations in QCD are intimately connected with chiral symmetry breaking and restoration through the axial anomaly, it is plausible that proximity to the critical point may amplify fluctuations of topological charge density (*160*, *161*).

Topological transitions in hot QCD matter, often described in terms of sphaleron processes, generate local chirality imbalance. If the rate or magnitude of such fluctuations is enhanced near the critical region, the resulting chiral chemical potential μ_5_ may become larger or more persistent. Because the CME current is proportional to μ_5_, increased topological activity would directly strengthen the anomaly-induced charge separation.

#### Interplay of medium properties and anomaly

The low-energy regime is therefore characterized by a nontrivial interplay between electromagnetic fields, medium transport properties, and QCD topology. Slower spectators and increased stopping favor a longer-lived magnetic field; enhanced conductivity supports electromagnetic induction effects; and possible critical fluctuations may increase the magnitude and persistence of chirality imbalance. Furthermore, the hydrodynamic evolution time of the medium becomes shorter at lower energy, with an earlier freeze-out time. This actually helps “lock in” the CME-induced charge separation signal, which primarily occurs at early times. Too long a fireball lifetime was found to reduce the charge separation because of normal viscous transport like charge diffusion (*153*). Together, these effects suggest that the CME signal could exhibit a nonmonotonic dependence on beam energy, potentially peaking in an intermediate energy window where both the magnetic field lifetime and topological fluctuations are optimized.

A quantitative assessment of these mechanisms requires realistic modeling of magnetohydrodynamic evolution, sphaleron transition rates at finite μ_*B*_, and the space-time overlap between the magnetic field and the deconfined phase. Nevertheless, the qualitative arguments outlined above provide a compelling motivation for focused experimental and theoretical investigation of anomalous transport in the low-energy regime of the BES.

### Open problems

Anomalous transport bridges high-energy physics, condensed matter, nuclear physics, and quantum information. The CME stands as a concrete realization of how spin, topology, and quantum anomalies shape macroscopic phenomena.

Open questions include the following:

1) Quantitative extraction of CME signal in heavy-ion collisions, 

2) Real-time simulations of anomalous magnetohydrodynamics, and 

3) Transport induced by gravitational and mixed anomalies. 

A century after the discovery of spin, anomalous transport reminds us that spin is not merely an intrinsic quantum number but a gateway to topology and collective dynamics.

## SUMMARY AND OUTLOOK

In this article, we have explored the role of spin in QCD across a wide range of physical regimes, from the internal structure of the nucleon to the collective dynamics of strongly interacting matter and anomaly-induced transport phenomena. Spin, found a century ago as an intrinsic quantum degree of freedom, emerges in QCD not merely as a property of elementary constituents, but as a dynamical feature of strongly interacting matter.

We began with the spin structure of the nucleon, reviewing the decomposition of the proton’s total angular momentum into the spin and orbital components of quarks and gluons. The proton spin puzzle revealed that the naive constituent quark model is insufficient and that gluon helicity and partonic OAM play essential roles. Modern theoretical frameworks based on gauge-invariant operator definitions, GPDs, and TMDs provide a multidimensional view of the proton’s spin structure, while ongoing and future experiments aim to complete this picture with increasing precision.

We then turned to spin phenomena in hot and dense QCD matter created in relativistic heavy-ion collisions. The observation of global hyperon polarization has demonstrated that the QGP is the most vortical relativistic fluid in which spin degrees of freedom couple to collective motion. The development of spin hydrodynamics and quantum kinetic theory has begun to establish a consistent framework for describing the dynamical evolution of spin, fluid vorticity, and their interplay both in and out of equilibrium in strongly interacting matter.

Last, we discussed anomalous transport and the CME as examples of how quantum anomalies connect chirality, topology, and macroscopic currents. In the presence of topological fluctuations of gauge fields and strong magnetic fields, the axial anomaly gives rise to dissipationless electric currents, which induce charge separation in heavy-ion collisions. Experimental searches, particularly in BES programs, continue to refine our understanding of the interplay between anomaly-driven signals and background correlations.

Together, these topics demonstrate that spin in QCD is a unifying concept linking hadron structure, relativistic many-body dynamics, and topological quantum phenomena. A century after its discovery, spin remains at the forefront of research, revealing new layers of structure in the strong interaction and continuing to illuminate the deep connections between symmetry, topology, and observable physics.

Looking ahead, the study of spin in QCD is poised to enter a new precision era. The ongoing JLab 12 GeV program, the COMPASS/AMBER program at CERN, and the forthcoming EIC will together provide unprecedented access to the multidimensional spin structure of the nucleon, enabling quantitative mapping of gluon helicity, OAM, and spin-orbit correlations at small Bjorken *x*. In heavy-ion physics, higher-statistics measurements and refined analysis techniques will further constrain anomalous transport signals and their backgrounds, while advances in spin hydrodynamics and real-time quantum simulations promise a more complete dynamical description of spin transport phenomena. On the theoretical front, lattice QCD, effective field theories, machine learning, and quantum information approaches are increasingly converging to illuminate the interplay between spin, entanglement, and topology in the QCD vacuum and in strongly interacting matter. These developments suggest that spin will continue to serve not only as a diagnostic tool but also as a fundamental organizing principle in our understanding of the strong interaction in the decades to come.
